# Rapid single-particle chemical imaging of nanoplastics by SRS microscopy

**DOI:** 10.1073/pnas.2300582121

**Published:** 2024-01-08

**Authors:** Naixin Qian, Xin Gao, Xiaoqi Lang, Huiping Deng, Teodora Maria Bratu, Qixuan Chen, Phoebe Stapleton, Beizhan Yan, Wei Min

**Affiliations:** ^a^Department of Chemistry, Columbia University, New York, NY 10027; ^b^Lamont-Doherty Earth Observatory of Columbia University, Palisades, NY 10964; ^c^Department of Biostatistics, Columbia University Mailman School of Public Health, New York, NY 10032; ^d^Department of Pharmacology and Toxicology, Ernest Mario School of Pharmacy, Environmental and Occupational Health Sciences Institute, Rutgers University, New Brunswick, NJ 08854; ^e^Department of Biomedical Engineering, Columbia University, New York, NY 10027

**Keywords:** optical microscopy, nanoplastics, Raman imaging, single particle analysis, Stimulated Raman Scattering

## Abstract

Plastics are now omnipresent in our daily lives. The existence of microplastics (1 µm to 5 mm in length) and possibly even nanoplastics (<1 μm) has recently raised health concerns. In particular, nanoplastics are believed to be more toxic since their smaller size renders them much more amenable, compared to microplastics, to enter the human body. However, detecting nanoplastics imposes tremendous analytical challenges on both the nano-level sensitivity and the plastic-identifying specificity, leading to a knowledge gap in this mysterious nanoworld surrounding us. To address these challenges, we developed a hyperspectral stimulated Raman scattering (SRS) imaging platform with an automated plastic identification algorithm that allows micro-nano plastic analysis at the single-particle level with high chemical specificity and throughput. We first validated the sensitivity enhancement of the narrow band of SRS to enable high-speed single nanoplastic detection below 100 nm. We then devised a data-driven spectral matching algorithm to address spectral identification challenges imposed by sensitive narrow-band hyperspectral imaging and achieve robust determination of common plastic polymers. With the established technique, we studied the micro-nano plastics from bottled water as a model system. We successfully detected and identified nanoplastics from major plastic types. Micro-nano plastics concentrations were estimated to be about 2.4 ± 1.3 × 10^5^ particles per liter of bottled water, about 90% of which are nanoplastics. This is orders of magnitude more than the microplastic abundance reported previously in bottled water. High-throughput single-particle counting revealed extraordinary particle heterogeneity and nonorthogonality between plastic composition and morphologies; the resulting multidimensional profiling sheds light on the science of nanoplastics.

## Significance

Micro-nano plastics originating from the prevalent usage of plastics have raised increasingly alarming concerns worldwide. However, there remains a fundamental knowledge gap in nanoplastics because of the lack of effective analytical techniques. This study developed a powerful optical imaging technique for rapid analysis of nanoplastics with unprecedented sensitivity and specificity. As a demonstration, micro-nano plastics in bottled water are analyzed with multidimensional profiling of individual plastic particles. Quantification suggests more than 10^5^ particles in each liter of bottled water, the majority of which are nanoplastics. This study holds the promise to bridge the knowledge gap on plastic pollution at the nano level.

Plastic pollution has been a rising global concern, with increasing plastic consumption every year (1). Microplastic contaminations have been identified to prevalently from almost everywhere in the environments and even human biological samples (2–4). Moreover, mounting discoveries suggest that the fragmentation of plastic polymer does not stop at the micron level but rather continues to form nanoplastics with expected quantities orders of magnitude higher (5). With engineered plastic particles with fluorescent dyes or metal labels, researchers have shown the possibility of nanoplastics crossing biological barriers and entering the biological systems (6–9), raising public concern on its potential toxicity (10).

Despite the urge to assess the concern, nanoplastics analysis remains challenging with traditional techniques. Unlike engineered nanoparticles prepared in laboratory as model systems, real nanoplastics in the environment are intrinsically label-free and have significant heterogeneity in both chemical composition and particle morphologies (11), which are likely to endure correspondingly different toxicity implications (12, 13). To address the existing knowledge gap on nanoplastics regarding their source, abundance, fate, and potential toxicity encoded in such a heterogeneous population, single-particle imaging with chemical specificity is undoubtedly essential to avoid informational loss from ensemble measurement. However, traditional single-particle chemical imaging techniques, namely FTIR or Raman microscopy, suffer from relatively poor instrumental resolution and detection sensitivity (14, 15), which limit their success in revealing the heterogeneity only at microplastic level (16, 17). Particle imaging techniques with nano-sensitivity for plastic particles, such as electron microscopy and atomic force microscopy, lack the crucial chemical specificity to distinguish different compositions (18, 19). Extensive efforts have been made; however, most techniques are still bound by the fundamental trade-off between sensitivity and specificity, a recurring theme in analytical science (15, 20). Single-particle imaging with chemical spectroscopy, recently demonstrated by AFM-IR and STXM (21–23), tends to have too low throughput (>10 min/µm^2^ with spectra for plastic identification) to quantify environmental micro-nano plastics with sufficient particle statistics. In summary, sensitivity, specificity, and throughput of single-particle analysis are the three crucial requirements to analyze nanoplastics in real-life samples.

Herein, we introduce a data science–driven hyperspectral stimulated Raman scattering (SRS) microscopy as a powerful platform of nanoplastics detection to meet the three requirements. SRS microscopy utilizes stimulated Raman spectroscopy as the imaging contrast mechanism and has found increasing utility in biomedical imaging (24–27). While SRS is often credited for speeding up regular Raman imaging by over 1,000 times (26–29), which enables fast identification of microplastics (30, 31), the utility for it to analyze nanoplastic remains to be explored. To maximize the sensitivity needed for single-particle detection, we adopted a narrowband SRS imaging scheme by focusing all the energy of the stimulating beam to target characteristic vibrational modes with the largest Raman cross-sections (32). We then showed that, both theoretically and experimentally, narrowband SRS imaging can enable the detection of nanoplastic as small as 100 nm. However, the limited spectral features from only the strongest vibrational signatures above the detection limit impose challenges on automated spectrum identification, which is essential for high-throughput plastic particle analysis. To address this fundamental sensitivity-specificity trade-off and unleash the full potential of hyperspectral SRS imaging, we devised a data-driven SRS-tailored spectral matching algorithm based on the spectral library of seven common plastic standards. The intrinsic chemical specificity from vibrational signatures in the shape of SRS spectroscopy is successfully recovered for automated polymer identification for nanoplastic detection with the help of the data science.

Equipped with this platform, we then studied micro-nano plastics in daily consumed bottled water as a prototype of a real-life sample. Individual particles for all seven plastic polymers from the library were identified, enabling statistical analysis of plastic particles with sizes down to 100 to 200 nm. The exposure to micro-nano plastics was estimated with a specified polymer composition. Integrating morphological information from imaging, multi-dimensional characterizations of individual plastic particles are reported, unveiling the all-around heterogeneities of plastic particles in a hidden micro-nano world encircling us.

## 1. SRS Imaging of Polystyrene Nanospheres with Single-Particle Sensitivity

SRS microscopy is well known to be orders of magnitude faster than regular Raman imaging (25, 26). The drastically higher imaging speed of SRS microscopy hence provides high throughput on particle imaging. However, whether high-speed SRS has a better detection limit than regular Raman and whether it can actually reach the single-particle sensitivity of nanoplastics are not obvious. A theoretical quantification is helpful to address the question in the first place. For a given major type of plastic polymer, we can estimate the mass of a 100-nm-diameter nanoplastics based on the plastic density and calculate the number of repeating units (i.e., constituting monomer) via its molecular weight. As shown in *SI Appendix*, Table S1, this number is around 10^6^ for most major plastic types, based on which we can further estimated the number of most abundant chemical bonds in a single plastic particle to be ~10^7^.

We can then theoretically explain why a 100 nm nanoplastic particle is difficult to be detected by conventional Raman microscopy. The spontaneous Raman cross-section of a typical C–H vibration is about 10^−29^ cm^2^. Hence, the spontaneous Raman cross-section of a 100-nm nanoparticle is 10^−22^ cm^2^. The laser waist area can be shrunk to about 2 × 10^−9^ cm^2^ under a high numerical aperture microscope objective. The probability of Raman scattering event per excitation photon is then (10^−22^ cm^2^)/(2 × 10^−9^ cm^2^) = 5 × 10^−14^. Assuming a moderately high laser power of 10 mW with a conventional 532 nm laser, which corresponds to an excitation flux of 3 × 10^16^ photons/s, and a rather long acquisition time of 100 ms (a small 128 × 128 image will take half an hour), only about 130 photons can be generated per particle in total via spontaneous Raman scattering. Considering the quantum yield of the entire instrument (including objective, filters, pinhole, spectrometer, and camera) typically is ~1%, roughly only 1.3 photons can be ultimately detected. Such a feeble signal can be easily overwhelmed by noise from other backgrounds such as autofluorescence.

**Fig. 1.**
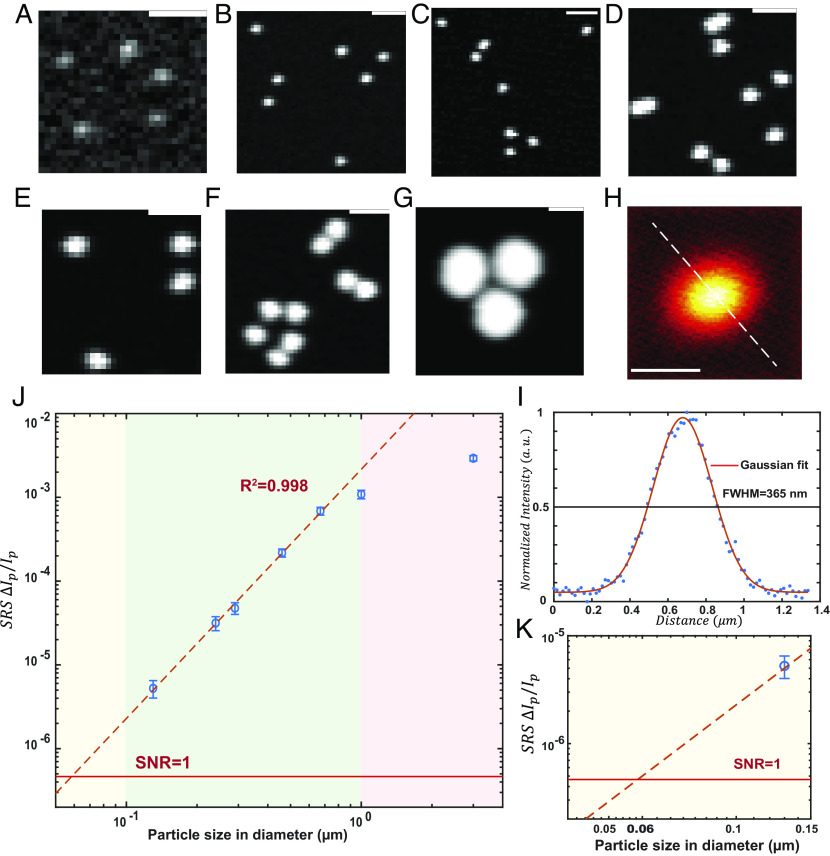
SRS imaging of standard PS micro-nano spheres for detection sensitivity and resolution characterization. (*A*–*G*) Representative SRS images (3,050 cm^−1^) of standard PS micro-nano sphere with different sizes: (*A*) 0.13 µm, (*B*) 0.24 µm, (*C*) 0.29 µm, (*D*) 0.46 µm, (*E*) 0.67 µm, (*F*) 1 µm, and (*G*) 3 µm. (Scale bar, 2 µm.) (*H*) SRS images of 0.24-µm PS nanosphere (3,050 cm^−1^) with 16 nm pixel size. (Scale bar, 0.5 µm.) (*I*) The normalized intensity distributions along the corresponding dash lines in Figure (*H*). (*J* and *K*) Linear dependence of the logarithm of stimulated Raman loss signals ( $\frac{\Delta I_{p}}{I_{p}}$ , measured at 3,050 cm^−1^) with the logarithm of particle size in diameter (µm). The red dashed line shows a linear fitting (R^2^ = 0.998) with a slope of 2.98. Error bars, mean ± SD. Red solid line indicates shot-noise-limited SRS detection limit where SNR = 1.

By employing an additional coherent Stokes laser, SRS amplifies the feeble scattering crossing section of a specific spectral mode (defined by the energy difference between pump and Stokes lasers) via quantum stimulation. When a pulsed narrowband Stokes laser is used (24, 33), the stimulated Raman enhancement factor can be maximized to more than 10^8^ (32, 34). The probability of a stimulated Raman scattering event per pump excitation photon then becomes 5 × 10^−6^, which is measured as a stimulated Raman loss experienced by the pump beam targeting C–H vibration. The noise of the pump beam under high-speed SRS microscopy acquisition (18 µs/pixel) is measured to be 5 × 10^−7^ (Fig. 1), which is about 10× lower than the expected stimulated Raman loss signal from a single 100-nm plastic particle. Thus, we predict that narrowband SRS shall break the detectability barrier of spontaneous Raman and bring a single nanoplastic particle into detection in just tens of microseconds.

We then experimentally verify the detection sensitivity using standard plastic particles. Polystyrene is one of the most common plastics widely used in daily life. Polystyrene particles of specified sizes are commercially available as analytical standards and have been routinely used as a model material to study micro-nanoplastics (35, 36). The Raman spectrum of polystyrene suggests a prominent peak at 3,050 cm^−1^ from aromatic C–H vibration on the phenyl ring (*SI Appendix*, Fig. S1), which can be selectively amplified for SRS imaging by tuning the difference of pump and Stokes beams to match this transition energy. Using commercial PS micro-nano spheres from 100 nm to 3 µm, we evaluated the detection sensitivity of our SRS microscope in imaging nanoplastics. To stabilize the particles during imaging, we embedded the diluted PS particles in agarose gel. As the particle size goes smaller, the residue of the water background around 3,000 cm^−1^ starts to dominate (*SI Appendix*, Fig. S2*a*), overwhelming the authentic spectrum of individual PS nanoparticles. To resolve this background issue for better imaging contrast, we substituted regular H_2_O with D_2_O to prepare the agarose gel (*SI Appendix*, Fig. S2*b*). Compared to H_2_O, the Raman spectrum of D_2_O is red-shifted to the silent region (2,200 to 2,800 cm^−1^, *SI Appendix*, Fig. S3), creating a background-free environment for probing C–H vibration.

SRS intensity of individual particles can be thereby measured from single-channel narrow-band imaging with high-throughput (~1,000 particles in one 51 × 51 µm FOV within 2 s, *SI Appendix*, Fig. S4). This imaging speed is orders of magnitude faster than other nanoplastic imaging techniques, such as AFM-IR and STXM (21, 23, 37). With the optical diffraction limit, the optimal spatial resolution of SRS microscopy is measured to be 365 nm (Fig. 1 *H* and *I*). With a spatial sampling of 200 nm pixel size for high-throughput imaging, individual PS nanospheres of above 500 nm can be discerned with their shape from the images (Fig. 1 *D–G*). When the size of the particles goes smaller than the diffraction limit (Fig. 1 *A*–*C*), the finite optical resolution renders the particle image a diffraction-limited pattern. Yet, the SRS intensity of a single particle can still be readily recognized down to 100 nm based on the diffraction limit pattern and the intensity distribution (*SI Appendix*, Fig. S5). Thus experimentally, we have shown that compared to regular spontaneous Raman, SRS imaging can offer orders of magnitude higher imaging speed/throughput and a superior limit of detection for nanoplastics analysis.

A linear relationship was observed between the logarithm of SRS signal ( ${\Delta I}_{p}/I_{p}$ ) and the logarithm of diameter for PS particles smaller than 0.7 µm (Fig. 1*J* and *SI Appendix*, *Supplementary Note* *3*). The trendline with a slope of 2.98 within the range indicates the SRS signal ( ${\Delta I}_{p}/I_{p}$ ) increase linearly with the particles’ volume, which scales in cubic as the particles’ diameters increase. When the particles’ size is enlarged to overfill the effective focal volume sequentially in first x, y, and later z dimensions (*SI Appendix*, Fig. S14), the linear dependency disappears. This good linearity (R^2^ = 0.998) is due to the fundamental linear dependency of the SRS signal on the concentration of the target analyte, providing powerful utilities in several aspects. First, the actual size of particles below the diffraction limit can be estimated based on the obtained calibration curve (*SI Appendix*, Fig. S16*a*), extending the size characterization limit. Second, with the known information on the plastic density, the same calibration curve can be transformed into a reference to deduce a particle mass out of a detected SRS nanoplastics image (*SI Appendix*, *Supplementary Note* *3* and Fig. S16*b*). Finally, taking an SNR of one as the threshold, the detection limit of our narrowband SRS microscope can be determined (Fig. 1*K*) to reach PS nanospheres down to 60 nm.

## 2. Fundamental Challenges on Chemical Identification of Nanoplastics with Hyperspectral SRS Imaging

**Fig. 2.**
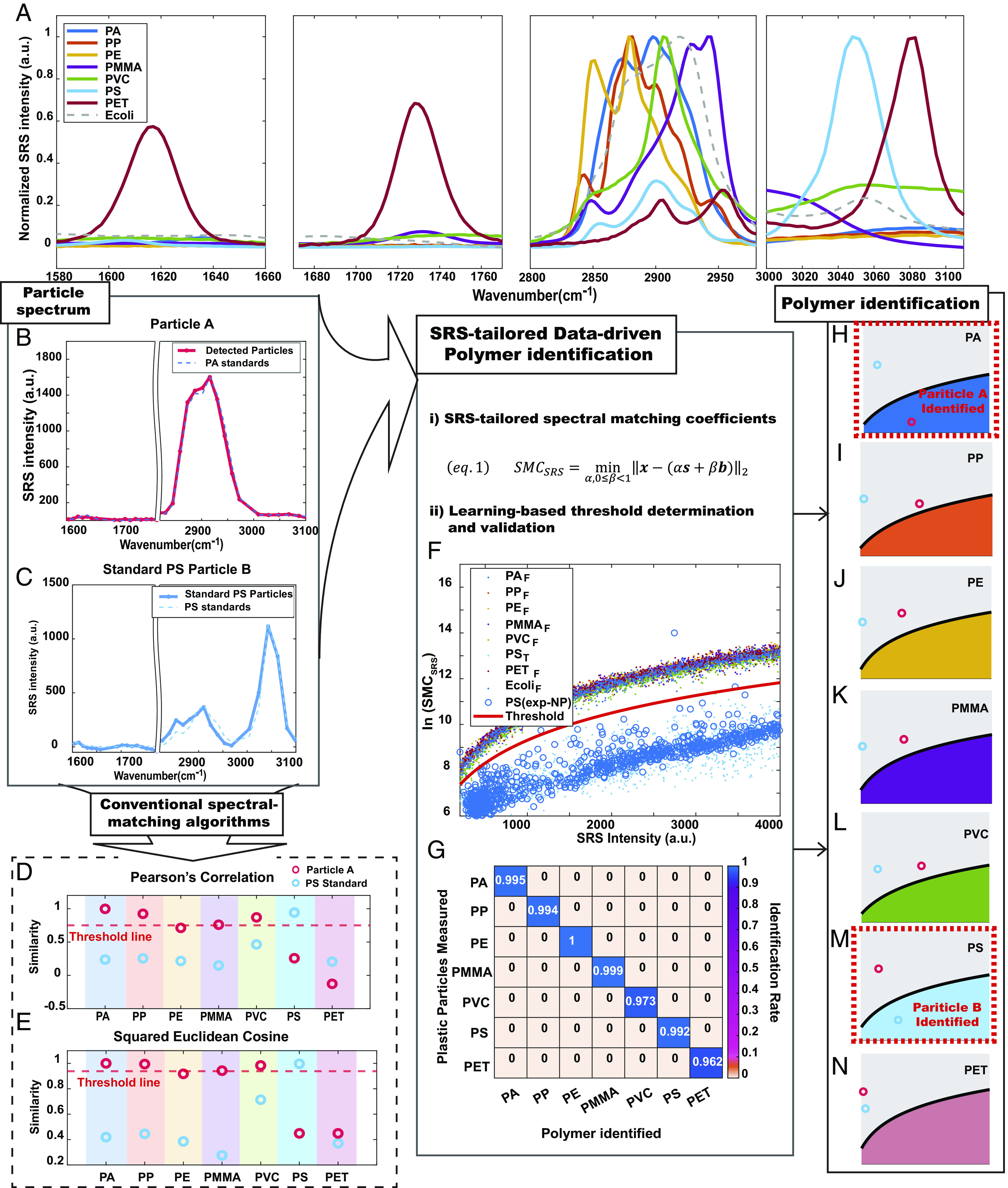
Recovering the chemical specificity for polymer identification with SRS-tailored data-driven spectral matching algorithms. (*A*) Normalized SRS spectra of plastic standards (PA, PE, PET, PMMA, PP, PS, and PVC) and a nonplastic standard (*E. coli*). (*B* and *C*) Examples of particle spectra: (*B*) particle A: PA microparticle (*C*) particle B: standard PS nanosphere. (*D* and *E*) Similarity quantification results for particles A and B from conventional spectral-matching algorithms: (*D*) Pearson’s correlation coefficients and (*E*) Squared Euclidean Cosine (SEC). The red dashed line indicates the threshold condition with 95% identification rate from the standard PS nanospheres. The same threshold condition creates elusive identification for particle A. (*F*) The learning process is indicated by the scatter plot of ln(SMC_SRS_) against SRS intensity α obtained from Eq. 1. Solid data points are from the synthetic dataset based on standards. The blue circular data points are experimental data from hyperspectral SRS imaging of PS nanospheres of three different sizes, which well colocalize with the points from the synthetic PS spectrum (light blue) with good separation from synthetic data from other chemical compositions (solid data points in other colors). The red solid line indicates the threshold line drawn for plastic polymer identification. (*G*) Confusion matrix for threshold condition evaluation based on experimental plastic particle measurement (*H*–*N*). Polymer identification results of the example particle A and particle B using SRS-tailored data-driven spectral matching algorithms. In each image of (*H*–*N*), the black line is the determined threshold from the learning process. The light blue circle from standard PS particle B is confirmed perfectly only with the PS matching scheme having the SMC_SRS_ value below the threshold line (Fig. 2*M*). The red circle from unknown particle A is unambiguously identified to be PA with only the PA matching scheme having the SMC_SRS_ value below the threshold line (Fig. 2*H*).

Nano-sensitivity solves the first-order issue to ensure the plastic particles are detectable. The chemical specificity of a technique is also crucial to identify plastics from other co-existing substances and further distinguishing plastic polymers from each other. Harnessing vibrational spectroscopy as imaging contrast, SRS microscopy, in principle, holds the demanded specificity for chemical imaging. Instrumentally, we perform hyperspectral SRS imaging via the spectral-focusing technique (38, 39). To best cover the characteristic strong feature of the plastic Raman spectrum (*SI Appendix*, Fig. S1) within the tuning range of the instrument (790 to 910 nm), we carefully choose 793, 804, 886, and 897 nm as four central wavelengths to include the strong and characteristic spectral features of C–H (unsaturated and saturated carbons, 3,110 to 2,800 cm^−1^), ester bonds (1,770 to 1,670 cm^−1^), and double bond vibration (1,660 to 1,580 cm^−1^) for better distinguishment between each plastic type. We constructed a small library by measuring the bulk SRS spectra of seven most common plastic polymers (Fig. 2*A*): polyamide 66 (PA), polypropylene (PP), polyethylene (PE), polymethyl methacrylate (PMMA), polyvinyl chloride (PVC), polystyrene (PS), and polyethylene terephthalate (PET) with fine spectral intervals (~3 cm^−1^).

Unlike bulk spectra measurement, single-particle imaging of nanoplastics requires a much smaller pixel size, longer integration time, and higher power for optimal signal-to-noise ratio. Therefore, due to the fundamental trade-off between detection sensitivity and specificity, it is nearly impossible to measure nanoplastics with such fine spectral intervals (hours of imaging time per FOV with increasing possibility of sample drifting and burning during the time). Moreover, the spectral resolution of a hyperspectral SRS microscope based on spectral focusing is typically 10 to 25 cm^−1^. For efficient hyperspectral imaging with a proper balance between throughput and spectral resolution, we further subsampled the spectra (*SI Appendix*, Fig. S6) with the spectral interval of ~15 cm^−1^, which is only slightly above the spectral resolution and yielded acceptable imaging throughput (~0.5 h per 0.2 mm × 0.2 mm FOV) for single-particle chemical imaging of nanoplastics.

High-throughput plastic particle analysis also requires automated spectral analysis for plastic identification. Spectral matching algorithms for automated chemical identification are prevalently adopted in microplastic analysis based on FTIR or Raman spectroscopy (40, 41). With thousands of particle spectra in need of analysis in a typical environmental study, manual plastic identification and counting are not only impossibly labor-intensive but also subjected to human bias (14, 40–42). Automated particle analysis helps to speed up the measurement, analyze more particles, as well as ensure ubiquitous and unbiased plastic identification. Understanding the need for automation in environmental science, we started with applying the classic library matching algorithms in FTIR and Raman analysis but found them not so compatible with narrow-band SRS hyperspectral analysis. Take a detected spectrum from particle A prepared from grinding the PA standard as an example (Fig. 2*B*). After spectrum pre-processing on background subtraction and data normalization, the spectrum of particle A clearly matches the SRS signature of polyamide. However, when measuring the spectral similarities of particle A to bulk plastic standards from the library using common spectral matching algorithms (42), such as Pearson’s correlation coefficient (PC) or squared Euclidean cosine (SEC) measurement, the identification results appears elusive (Fig. 2 *D* and *E*). In a real-life sample analysis, there should be no premise to assume particle A should belong to any standard plastics in the library, which means a yes or no judgment has to be made independently for each plastic standard based on a given threshold. The common threshold employed in FTIR or spontaneous Raman analysis of microplastics is the similarity measurement above 0.7, which is clearly too low to identify Particle A. Since PS nanoparticles are available as model standards, we first try to study the similarity threshold of each algorithm for nanoplastics analysis under hyperspectral SRS imaging. The similarity threshold can then be determined based on the quartile of identifying at least 95% of the PS particles (similarity index above 0.75 for PC, and similarity index above 0.94 for SEC). However, the challenging part of making a binary identification judgment remains in the case of particle A as similarity measurements from three plastic polymers (PA, PP, and PVC) are very close in number and all above the threshold (Fig. 2 *D* and *E*). Note that one cannot simply pick the best score among all the standards because it is totally possible for A to be nonplastic materials in real sample analysis. In fact, if we simulate the possible nonplastic SRS spectra based on the model standard spectrum of biomass represented by *E. coli*, over 95% of them will have similarity measurements against PA standard over the given threshold for both two algorithms (*SI Appendix*, Fig. S12 *a* and *b*).

We reflect that the main reason underlying the above difficulty stems from the trade-off between detection sensitivity and specificity. Emphasizing the chemical specificity, spontaneous Raman spectroscopy, or other broadband coherent Raman microscopy can cover an extended spectral window (>1,000 cm^−1^) by distributing the optical power among a large number of Raman vibrational modes. The rich spectral information can enable chemical identification with simple algorithms but comes with the cost of over thousand times compromised detection sensitivity under a limited pixel dwell time (43–45). However, in the context of nanoplastics analysis, detecting the particle signal is the premise before chemical identification from the vibrational spectrum. With the aim of measuring as small plastic particles as possible under practical throughput, eventually, only the strongest Raman features will be detectable with reasonable SNR. For most plastics, which are organic polymers by nature, the strongest Raman signatures reside within the limited C–H vibration window. In this case, specific chemical identification requires the algorithms to precisely capture the shape feature within the restricted spectral window, which is beyond the capacity of conventional spectral matching algorithms. Moreover, the inevitably compromised and circumscribed signal-to-noise ratio when imaging diminutive nanoparticles create further challenges in spectral interpretation for robust chemical identification. Therefore, new methods are demanded to address the specificity challenge imposed by the SRS instrumentation that enables unprecedented sensitivity in imaging nanoplastics.

## 3. Data-Driven SRS-Tailored Spectral Matching Algorithm Recovers Chemical Specificity

Harnessing data science, we aim to develop algorithms to interpret the shape of detected SRS features and retrieve the chemical specificity for polymer identification. First, an SRS-tailored spectral matching coefficient (SMC_SRS_) is developed as an indicator to quantify spectral similarity with minimized noise interference (Fig. 2, eq. 1). SMC_SRS_ uses an optimization algorithm that considers the detected SRS spectrum x originating from scaling (intensity factor α ) the normalized bulk standard spectrum s, plus a certain background contribution at the imaging condition ( βb , 0≤β < 1). The fitted spectrum ( αs+βb ) was compared with the detected particle spectrum x to find the minimum possible spectral distance as SMC_SRS_. The smaller SMC_SRS_ value indicates a higher spectral similarity to the corresponding standards. This indicator SMC_SRS_ provides several advantages for the purpose of detecting nanoplastics. The optimization algorithm considers all spectral points simultaneously, which reduces the direct influences induced by the noise on each particular spectral point. The fitting process leverages the reliability of the similarity measurement. In addition, the outcome of the measurement is interpretable. The well-defined intensity factor α and background factor β can indicate the contribution from each spectral component (the particle and the surrounding backgrounds). Finally, the spectral distance measurement provides metric similarity evaluation.

With the spectral similarity quantified in this refined way, we returned to face the challenge of making a nonarbitrary binary judgment for polymer identification. We planned to develop a learning-based method to determine the previously elusive binary threshold for the identification of all plastic polymers. Our premise is that if we can measure the nanoparticle spectra for all types of plastics within the library, we shall be able to learn from the data and draw the correct boundary for identification based on the distribution of the particles with known identities. However, in reality, only PS nanospheres are commercially available to us with well-characterized chemical composition and nano sizes. Without reliable ground truth from other polymer nanoparticles, we have to seek alternative ways to gather the massive information needed for rigorous threshold determination.

Inspired by the increasing utilities of synthetic data in AI (46), and the growing involvement of data science in SRS microscopy (47–49), we realized that we could simulate the experimental SRS spectra of nanoplastics from the bulk standard spectra to serve as a training dataset (i.e., synthetic data). Based on our understanding of the SRS instrumentation, we proposed a model, where there are two main sources of noise in a typical hyperspectral SRS spectrum: one is fundamental noise on the SRS intensity as in a shot-noise-limited scenario, which can be easily read out from the same SRS image; the other is the frequency uncertainty imposed by the SRS instrumentation, where both the laser profile and the moving delay stage can result in fluctuation of the actual frequency excited in each measurement around the preset spectral points. Assuming the fluctuation follows a Gaussian distribution, we used PS nanospheres as the standard model to investigate the fluctuation range and found an impressive consistency in SMC_SRS_ calculation from the synthetic spectra and measured spectra of PS nanoparticles (*SI Appendix*, *Supplementary Note 2* and Fig. S10). The combinatory nature of noise origins explains the dependency of the SMC_SRS_ value on the intensity of the spectrum ( α ), as suggested in the simulation and validated by the experiment (Fig. 2*F*).

Applying the same model for all standards in the library, we generated a synthetic dataset containing the possible SRS spectra for nanoplastics of each polymer in the plastic library. A nice separation of the SMC_SRS_ value appears between the spectra of particle X ( X=R , R is the correct identity of standard polymer) and spectra of particle X ( X≠R ) in all scatter plots (*SI Appendix*, Fig. S11). With the massively generated synthetic data points, a logarithmic function was fitted according to the trend of the scattered points as the threshold line for polymer identification (*SI Appendix*, *Supplementary Note 2* and Table S2).

We first evaluate the identification performance by simulating another synthetic dataset from all standards in the library as testing data. Compared with conventional spectral matching algorithms, the SRS-tailored developed shows minimal false positives in plastic identification (*SI Appendix*, Fig. S12). No more than 0.5% of nonplastic spectra (simulated from *E. coli*) is misidentified as a hit for any plastic types in the library (*SI Appendix*, Fig. S12*c*), which is a drastic improvement from over 97% using conventional spectral matching algorithms (*SI Appendix*, Fig. S12 *a* and *b*). False positive between polymers of similar SRS spectrum is also much reduced with the maximum to be around 5% PA misidentified as PP (*SI Appendix*, Fig. S12*c*). The same number is also as high as over 97% if PC or SEC are used as similarity measurements with the determined thresholds (*SI Appendix*, Fig. S12 *a* and *b*).

To further address the possible rare cases where a particle is identified as hits for more than one polymer in the library, the chemical identity of the corresponding particle will be assigned to the polymer with the smallest SMC_SRS_ value. With the established spectral identification workflow, an over 96% identification rate can be achieved with a false positive rate below 1% for all polymers in the library (*SI Appendix*, Fig. S12*d*). Since PS nanosphere was the only available nanoplastic standard, the experimental validation of the workflow is based on the imaging of the corresponding microplastics prepared from grinding the polymer standards with the cryo-mill. Hoping to mimic a similar level of spectral variation to the best extent, the imaging condition is adjusted accordingly to match the signal-to-noise ratio of nanoplastic measurement. Finally, we confirmed the same identification rate of over 96% in the experimental particle measurement with no observed plastic particles misidentified as other polymers within the library (Fig. 2*G*).

Development of this data-driven algorithm allows for the identification of each plastic polymer with distinct vibrational features in a restricted spectral window, thus retrieving the required chemical specificity for automated spectral identification. Revisiting the identification of particle A and standard PS nanosphere B, we can correctly identify both particle A and particle B across the library to be PA and PS (Fig. 2 *H–N*), with SMC_SRS_ well captures the shape differences missed by conventional algorithms and threshold learned from the data-driven study. Coupling the mindset from data science with advanced measurement science, we finally overcome the fundamental sensitivity-specificity trade-off for high throughput hyperspectral SRS analysis. Superb nano-sensitivity from narrow-band SRS amplification and chemical specificity with robust chemical identification are simultaneously accomplished to fill the missing void in tools for nanoplastics analysis.

## 4. Developing Workflow for Micro-Nano Plastic Detection from Bottled Water

With the platform established, we moved on to apply the utility to study micro-nano plastics from real-life samples. Microplastics have been widely found in human foods (50), drinks (51), and product packaging (52–55), among which bottled water is of particular interest for being an important source of microplastics to be ingested in daily life (56–59). Limited by the sensitivity-specificity trade-off in analytical science (*SI Appendix*, Fig. S18*b*), the literature knowledge is constrained to microplastics in bottled water (*SI Appendix*, Table S4) (19, 60–62), leaving the nanoplastics mostly uncharted. So far, only ensemble characterizations using combinations of techniques are reported to analyze the aliquots of concentrated nanoparticles from bottled water. Information is demanded to address the intrinsic heterogeneity of nanoplastics contamination at a single-particle level (*SI Appendix*, Fig. S18*a*) (63, 64). Here, we report a concise workflow for comprehensive micro-nano plastics characterization enabled by rapid single-particle chemical imaging with nano-sensitivity by SRS microscopy. Rich information can be acquired from a single measurement to achieve simultaneous characterization of chemical composition and morphology, enabling multi-dimensional statistics through high-throughput single-particle analysis.

Filtration is one of the most common methods to collect particles above certain sizes onto a membrane surface. It would be highly preferable for analyzing real-world samples if the collected membrane is directly compatible for SRS imaging. Aluminum oxide membranes have minimal background in the target spectral window and have shown good compatibility with vibrational spectroscopy. The seemingly opaque aluminum oxide membrane can be easily transformed into a transparent imaging window by applying heavy water to reduce refractive index mismatch. This resulted in transmissive SRS imaging with acceptable signal retention (~70% of the original sensitivity, *SI Appendix*, Fig. S7 *b* and *c*). Embedding the particles on the membrane surface in situ with agarose gel prepared with D_2_O further enabled stationary SRS imaging of individual particles with minimal imaging background. In this way, a concise sample preprocessing is enough for high-quality SRS imaging of the original filtration membrane (*SI Appendix*, Fig. S7*a*), avoiding undesirable sample loss or contamination in any complicated sample drying or transferring processes.

**Fig. 3.**
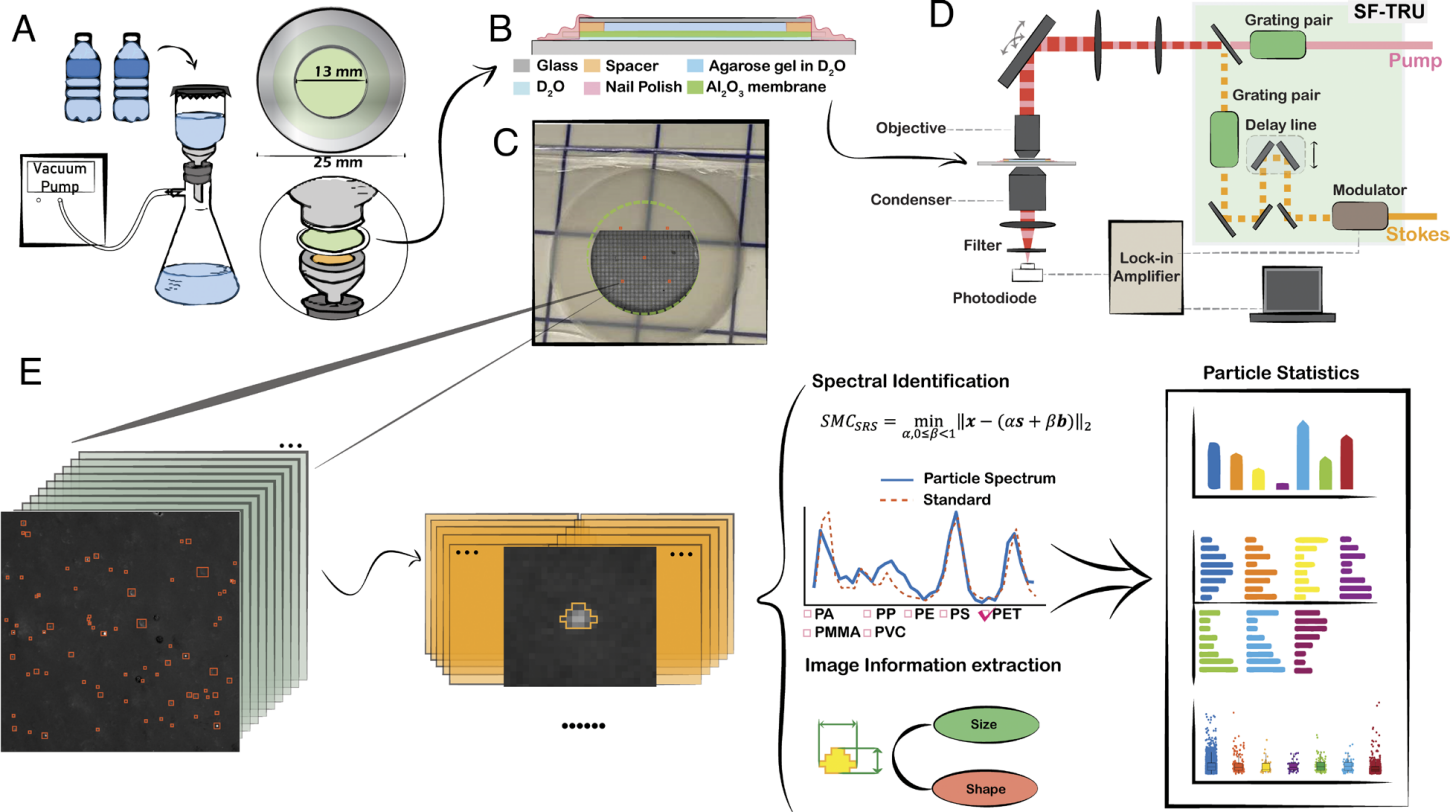
Detecting micro-nano plastics in bottled water: sample preparation, SRS imaging, and data analysis. (*A*) Scheme of the filtration setup for collecting micro-nano plastic particles from bottled water. The particles from the two bottles of water samples are concentrated onto a circular area (d = 13 mm) at the center of the membrane following the procedure described in Supplementary information. (*B*) Scheme of membrane sandwiching to prepare transparent membrane samples for SRS imaging. The obtained sample (Fig. 3*C*) is then mounted onto the microscope (Fig. 3*D*) for hyperspectral SRS imaging. (*C*) The obtained transparent membrane sample superimposed with a fluorescence image of the standard fluorescent PS particles collected on the membrane illustrates the uniform particle distribution on a circular surface in the center of the membrane (*SI Appendix*, *Supplementary Note 5*). (*D*) Scheme of the SRS microscope. (*E*) Scheme of automated plastic particle identification. The preprocessed stacks of hyperspectral SRS images are analyzed by a MATLAB script for automated plastic particle identification. For each on-resonance image for the target plastic polymer, detected particles are segmented as regions of interest (ROIs) to extract the chemical and morphological information for analysis. The SRS spectrum is extracted in each particle/ROI by intensity measurement across the hyperspectral image stack. For particles with SRS peaks in the correct corresponding spectral window, spectral similarity to the target plastic standard is quantified by calculating SMC_SRS_ with the threshold condition applied to make the plastic identification judgment. Morphological information such as size and shape is extracted in the course of image analysis, and statistical pictures composed by each identified individual plastic particle are created subsequently.

**Fig. 4.**
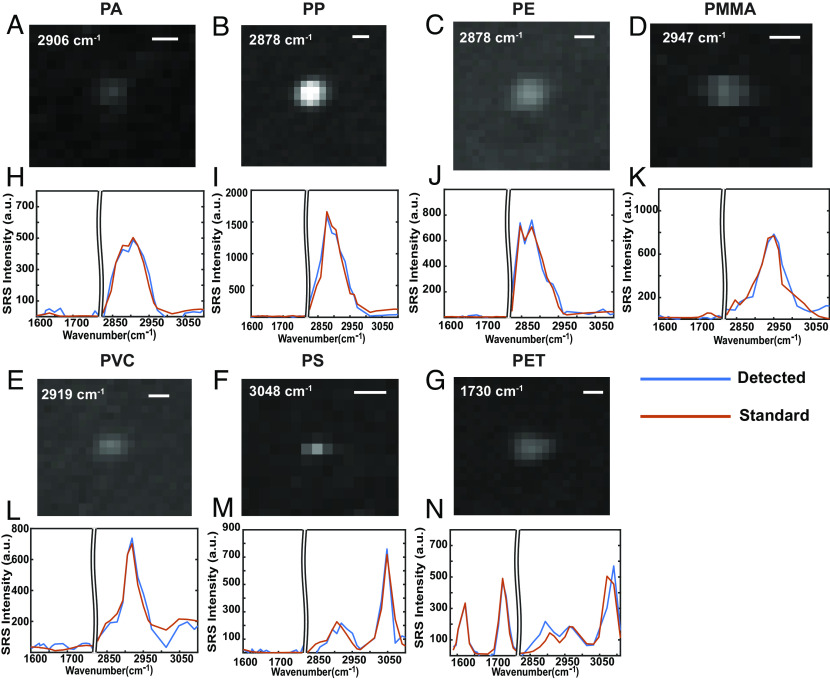
Individual micro-nano plastic identified for each target polymer from bottled water. (*A*–*G*) Representative SRS images of fine plastic particles detected for each polymer: (*A*) polyamide, (*B*) polypropylene, (*C*) polyethylene, (*D*) polymethyl methacrylate, (*E*) polyvinyl chloride, (*F*) polystyrene, and (*G*) polyethylene terephthalate. (Scale bar, 0.6 µm.) Most of these particles are below 1 µm. (*H*–*N*) Corresponding SRS spectra of the detected plastic particles. The blue lines are the spectra of detected particles. The orange lines are the matched spectra from the plastic standards.

The established workflow for analyzing micro-nano plastics exposure from bottled water with hyperspectral SRS imaging is presented in Fig. 3. For each sample, five or more fields of views (FOVs) were randomly sampled within the collecting area for hyperspectral imaging under SRS microscopy (Fig. 3*D*). In each FOV, micro-nano plastics were detected by an integrated data analysis workflow that automatically performed the particle segmentation and plastic identification with the developed algorithms and validated threshold conditions. Morphological and chemical information of each individual plastic particle obtained from the hyperspectral SRS images was then combined to provide high-dimensional profiling (Fig. 3*E*). Following the procedure, we analyzed bottled water from three different brands acquired at the same time from a large retailer. With no access to plastic-free water in the lab (*SI Appendix*, *Supplementary Note 6*), the Anodisc filters are prepared and measured in the same way as blank control. In the results, we were able to detect individual particles for all seven plastic polymers in the library unambiguously by spectral matching with their corresponding bulk standards (Fig. 4), demonstrating the powerful plastic identification capability of our data-driven hyperspectral SRS imaging platform.

## 5. Multidimensional Profiling of Micro-Nano Plastic in Bottled Water

Quantification from single-particle images with identified plastic polymer composition provides multi-dimensional information to build the analytical panorama of underexplored nanoplastics in bottled water.

**Fig. 5.**
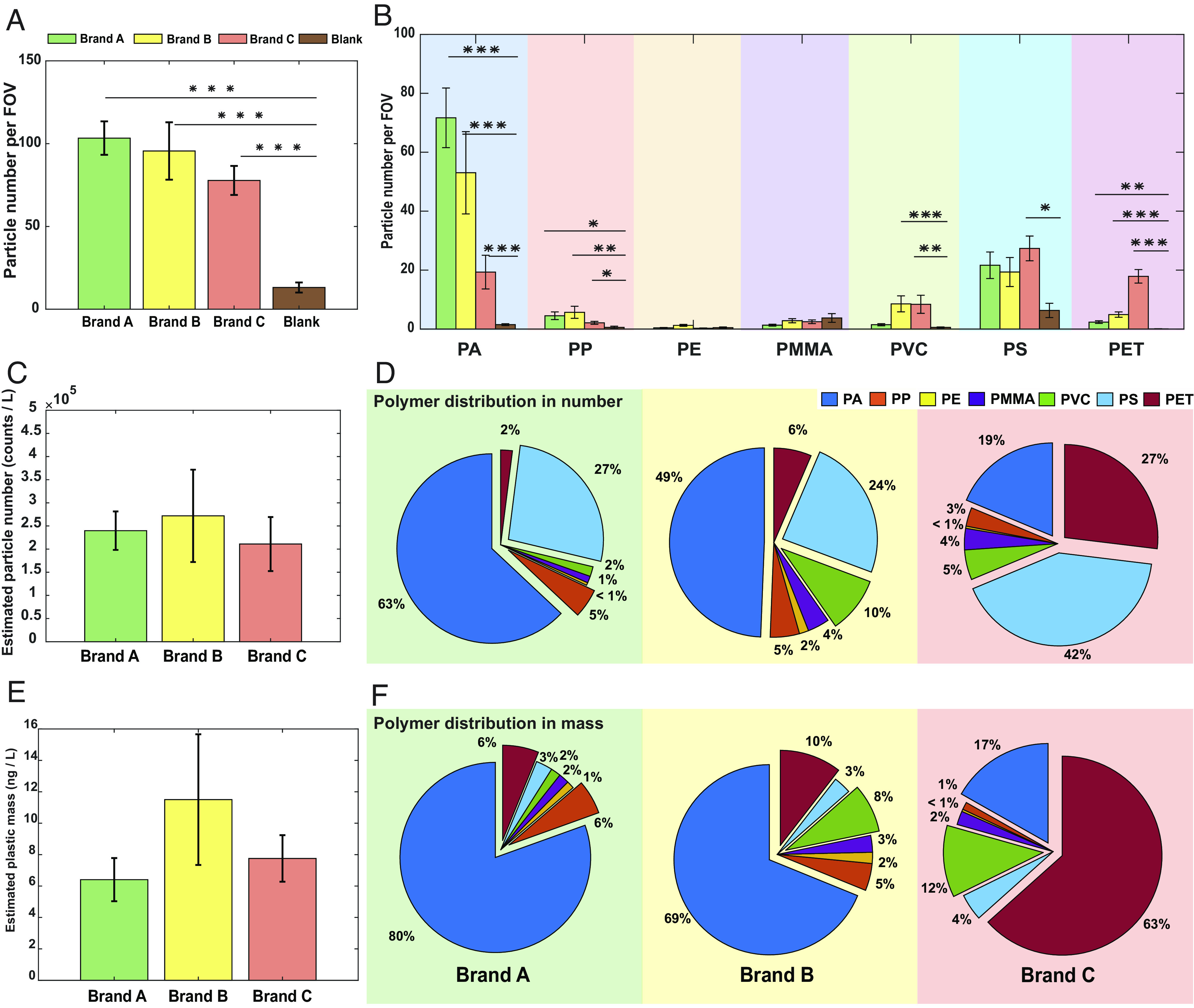
Quantification of micro-nano plastic exposure from bottled water. (*A*) Averaged number of plastic particles detected per field of view. Error bars, mean ± SEM. (*B*) Averaged number of particles for each plastic polymer detected per field of view. Error bars, mean ± SEM. Statistically significant differences were determined using generalized linear mixed model analysis with Bonferroni correction. *P < 0.05, **P < 0.01, and ***P < 0.001. (*C*) The number of plastic particles estimated in 1 L of bottled water. Error bars, mean ± SEM. (*D*) Number proportion of each plastic polymer measured in each brand of bottled water. (*E*) Mass of plastic particles estimated from SRS intensity in 1 L of bottled water. Error bars, mean ± SEM. (*F*) Mass proportion of each plastic polymer measured in each brand of bottled water.

Number quantification through particle counting suggests that on average, 78 to 103 plastic particles were identified in each FOV (0.2 mm × 0.2 mm) for three different brands, which was significantly higher (*P* < 0.001) than the blank samples (Fig. 5*A*). Assuming a uniform distribution of micro-nano plastic particles on the surface of the membrane region (*SI Appendix*, *Supplementary Note 5*), we can make an estimation for the micro-nano plastic exposure from bottled water. We estimate that there are on average about 2.4 ± 1.3 × 10^5^ plastic particles ingested from every liter of bottled water measured from different brands(Fig. 5*C*). Individual particles of each type of polymer are analyzed separately to reveal chemical heterogeneity. Within the library, PA, PP, PET, PVC, and PS are found likely to play a significant role in micro-nano plastics exposure from bottled water (Fig. 5*B*). The exact chemical composition of the micro-nano plastics varied from brand to brand, but PA seem to be the common major contributors in number among all the three brands we analyzed.

Harnessing the linear relationship between SRS intensity and the amount of analytes within the focal volume, we are also able to provide an estimation of exposure in mass besides particle number. The mass calibration curve can be estimated for each polymer out of density and relative SRS intensity from the linear relationship obtained by standard PS nanospheres (*SI Appendix*, Fig. S16). Integrated intensity within the region of interest for each particle is thus converted to mass (Fig. 5 *E* and *F*). The estimated micro-nano plastic exposure in mass is calculated to be at the level of around 10 ng/L. Analyzing the chemical composition in mass, we find unneglectable differences between contribution quantified by mass and contribution by number. Take the results from Brand C as an example. The PS nanoplastics though dominated in particle number, only account for a minor portion of the mass. Instead, PET becomes the major contributor together in mass. Such seeming disparity highlights the potential misunderstanding of plastic composition from collective particle characterization, which originated from the heterogeneous nature of micro-nano plastics from real-world samples.

**Fig. 6.**
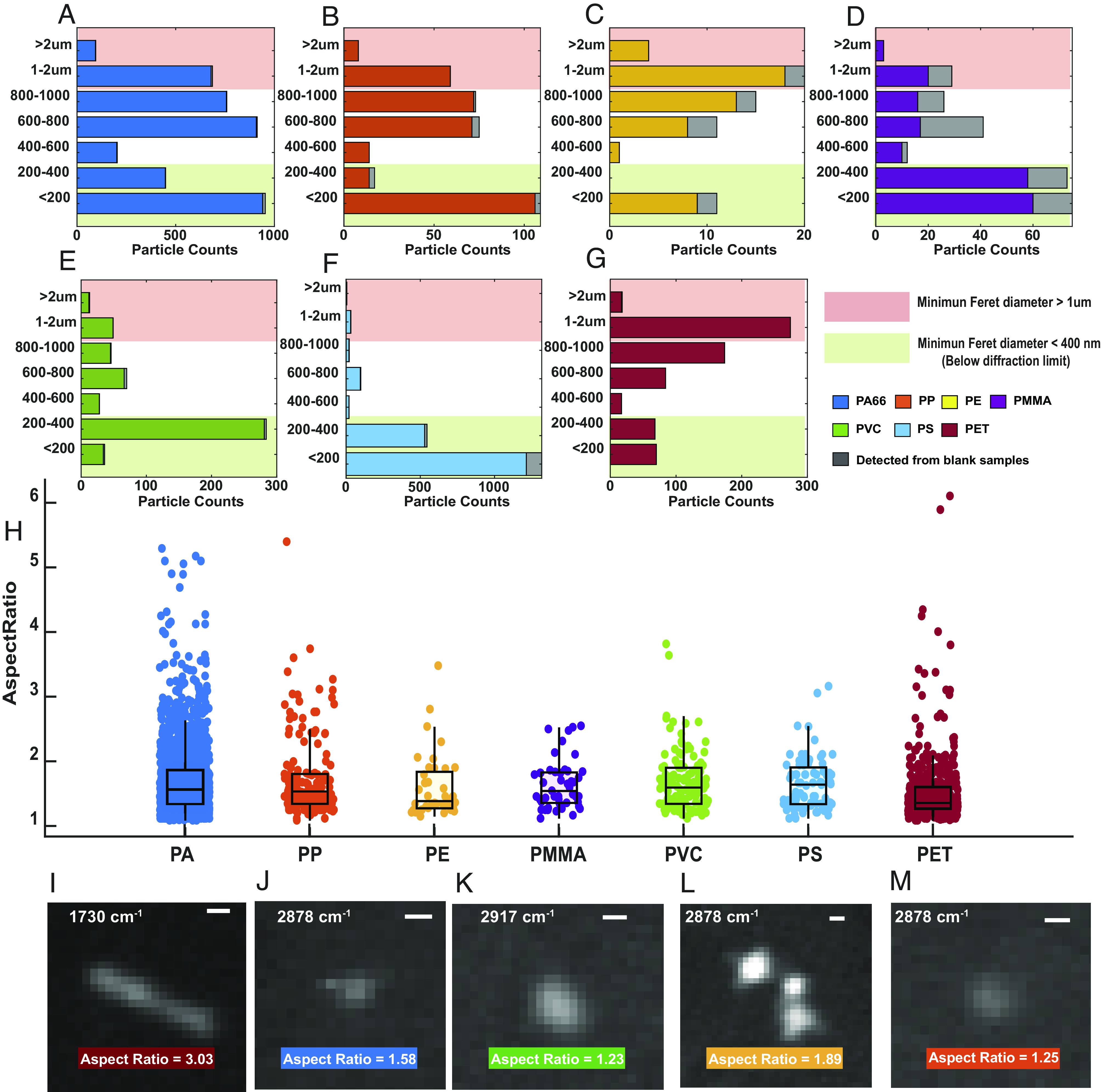
Statistical profiles of particles’ size and shape for each plastic polymer found in bottled water. (*A*–*G*) Size distribution of the detected particles for each plastic polymer: (*A*) polyamide, (*B*) polypropylene, (*C*) polyethylene, (*D*) polymethyl methacrylate, (*E*) polyvinyl chloride, (*F*) polystyrene, and (*G*) polyethylene terephthalate. The red shaded area indicates the microplastics. The green shade area indicates the particles with sizes below the optical resolution of SRS microscopy, which are detected in a diffraction-limit pattern. For particles with size above the diffraction limit, the size of the particles is measured by minimum Feret’s diameter. For particles detected as diffraction-limited patterns, the actual size of the particles is estimated from SRS intensity harnessing the linear relationship between SRS intensity and the volume of the nanoparticles, assuming that nanoplastics exist as a solid sphere. (*H*) Shape distribution of the detected particles for each plastic polymer measured by aspect ratio. (*I*–*M*) Representative SRS images of plastic particles with various shapes indicated by different aspect ratios. *SI Appendix*, Fig. S9 shows the corresponding SRS spectra. (Scale bar, 0.6 µm.)

Morphological characterization of individual particles enabled by SRS microscopy directly reveals another dimension of particle heterogeneity. Statistical analysis of particle size and shape from the images of individual micro-nano particles with well-defined identities is reported. When measuring the size distribution, we are able to characterize particles below the diffraction limit by extrapolating the size from the intensity reading (assuming the particles as solid spheres) and by using the linear relationship between the volume of the particles and SRS signal as calibration (*SI Appendix*, *Supplementary Note 3*). As a result, we find that plastic particles of different chemical compositions actually have different size distribution patterns (Fig. 6 *A–G*). The direct observation of the particle heterogeneity here provides a natural explanation of chemical compositional differences observed from mass or number measurement. Take PS and PET as an example: the size distribution of PS particles centers around 100 to 200 nm, whereas PET particles tend to have a size distribution that nears 1 to 2 microns, which explains why PET is a more significant component when measuring in mass while PS clearly dominates when counting the number of particles (Fig. 5 *D* and *F*).

The shape is another important morphological feature that matters as a critical aspect of nanotoxicity. Studies have shown that shape plays a role in determining the cellular uptake of micro-nano particles (65, 66). SRS images of plastic particles confirmed the existence of shape diversity for micro-nano plastics in bottled water. To account for the shape of plastic particles in a statistical manner, we measure the aspect ratio of individual particles above the diffraction limit (Fig. 6*H*). The aspect ratio is widely acknowledged in nanotoxicology studies (67, 68). The aspect ratio of the plastic particles detected ranges from 1 to 6, and the average aspect ratio for particles is around 1.7. Fig. 6 *I–M* provides a pictorial view of how the aspect ratio is related to the particle shape. Particles with an aspect ratio of above 3 are most likely to be fibrous in shape, while particles with an aspect ratio of below 1.4 will be largely spherical. Shape variation on plastic particles has been found in all polymers detected, confirming the widely recognized idea that real-world micro-nano plastics have diverse morphological prosperities. This dimension is hard to be resembled by engineered polymer nanoparticles commonly studied in research laboratories, and the toxicological consequences pertaining to real-life plastic particle exposures and their differing physicochemical properties (i.e., size, shape) have yet to be determined.

## 6. Discussions and Conclusions

By developing the data-driven hyperspectral SRS imaging platform for micro-nano plastic analysis, we describe a methodology to improve nanoparticle detection sensitivity and polymer identification specificity, which has allowed us to start to address the long-lasting knowledge gap of nanoplastics. We estimate that the exposure to the micro-nano plastics from regular bottled water was at the level of 10^5^ particles per liter, which is two to three orders of magnitude more than the previously reported results merely focusing on large microplastics (*SI Appendix*, Table S4) (58, 59, 61, 69, 70). As it pertains to the estimation of human exposure, these values are substantially higher than those currently reported in the literature (56, 71), which is a result from the newly detected nanoplastic fraction of plastic particulate. The tiny particles previously invisible under conventional imaging actually dominate in number and account for ~90% of the entire population of plastic particles detected. The remaining 10% identified as microplastics have a concentration of around 3 × 10^4^ particles per liter (*SI Appendix*, Fig. S17), with the majority of them in the size below 2 µm. Larger particles (>2 µm), which are easier to identify under regular optical microscopy, are in the same order of magnitude as the reported microplastic analysis depending on the detection limited reported based on different technologies (*SI Appendix*, Fig. S17 and Table S4). Our results confirm the plastic fragmentation beyond the micron level by unambiguously detecting nanoplastics in real-life samples. Similar to many other particle size distributions in the natural world, there are substantially more nanoplastics, despite being invisible or unidentified under conventional particle imaging techniques, than previously counted large micron ones. This population of nanoplastics can be easily overlooked in mass quantification as well since nanoparticles with smaller sizes contain cubic-less substances. However, given the capability of these nanoplastic particles to cross the biological barrier, nanoparticles, despite the seemingly trivial contribution to the mass measurement, might play a predominant role in terms of toxicity evaluation (72, 73).

We also find many detected particles present SRS spectra that do not match any of the standards. In fact, our small library of seven plastic polymers can only account for roughly about 10% of the total particles/dots imaged under SRS microscopy. A similar level of identification rate is reported in the microplastic analysis in bottled water using vibrational microscopy, indicating the complicated particle composition inside the seemingly simple water sample (*SI Appendix*, Table S4). In this sense, if we assume all detected organic particles originate from plastics [the same assumption entailed by the quantitative result from SEM-EDX or Nile Red staining (19, 74)], the micro-nano plastic concentration could be as high as 10^6^ particles per liter. However, the common existence of natural organic matter certainly requires prudent distinction from spectroscopy with polymer specificity. Moreover, careful investigation of unidentified particles suggests other aspects that further increase the complexity of identifying chemical composition. For example, some particles exhibit identical features to the characteristic two peaks (C=O ester bond: 1,730 cm^−1^; C=C double bond: 1,615 cm^−1^) of the PET in the fingerprint region but present a great variety of vibrational peaks in the high-frequency C–H region (*SI Appendix*, Fig. S8 *a–d*). It is unlikely for a polymer material distinct from PET to display both the C=O and C=C vibrational signatures that perfectly match the standard PET spectrum. A more plausible explanation is that they are small heteroaggregates containing PET and other components, with their SRS spectrum being the superposition of the spectrum from each component. Indeed, for some larger ones, we can even capture the spatial chemical heterogeneity within the aggregates (*SI Appendix*, Fig. S8 *a*, *e*, and *i*). The possible formation of heteroaggregates between nanoplastics or other natural organic matter has long been recognized as a potential challenge in the analysis of nanoplastics and may influence toxicological outcomes within a biological exposure (11). Direct visualization of such heteroaggregates here in real-world samples supports such concerns. For other possible heteroaggregates formed without PET, rigorous identification will require expanding the spectral library and advancing analytical algorithms for SRS microscopy or other vibrational imaging techniques with extended spectral windows to address challenges imposed by massive particle heterogeneity (27, 75, 76).

Another important insight is that the particle size distribution varies with the different chemical compositions, suggesting an interconnection between particle morphology and chemical composition. The observed nonorthogonality between plastic composition and particle morphologies challenges the conventional assumption for micro-nano plastics characterization from ensemble measurement. Take the result from brand C analysis as an example, ensemble measurement of micro-nano plastics might suggest that the major substance is PET from compositional analysis and most of the plastic particles have sizes below 500 nm from the morphological analysis. Assuming the two dimensions as being independent properties, people might have an impression that most of the plastic particles in the bottled water from brand C should be PET particles with a size below 500 nm. However, our result from single-particle analysis presents a clear disparity: the sample turns out to contain a small number of PET particles of about micron size and a large number of PS particles with size below 500 nm.

Such nonorthogonality might provide valuable information to understand, trace, and eventually prevent possible sources of micro-nano plastic contamination. Specifically in drinking water production, plastic contamination is confirmed in every step from the well to the bottle (77). The discovered size differences among different plastic polymers might indicate precious information about contamination sources during water production. For example, PET and PE, which are used as the packaging material for bottled water for all three brands we analyzed, have similar size distribution patterns, with a major population of micron sizes compared to other polymers. A possible explanation is that some particles of this kind are newly released from the bottle package during transportation or storage, which are retained faithfully in the water sample. Other polymers such as PA, PP, PS, and PVC, which are not the packaging material but also identified with significant numbers, are most likely introduced before or during water production. PP and PA, which share the same broad distribution of sizes, are widely used as equipment components or coagulant aids in water treatment (78). Particularly, PA is the most popular membrane material used in reverse osmosis (79), which is a common water purification method shared by all three brands. PVC and PS, which have a unique size distribution favoring small nanoplastics, might indicate a contamination source even earlier. PVC is identified to be the most abundant polymer type in raw water from microplastic analysis (77). PS is known to be used as backbone material for ion exchange resins in water purification (80). It is possible large particles of PVC or PS get removed by the RO membranes in the later step of the water treatment, leaving mostly nano populations.

Lastly, the interconnection between particle morphology and chemical composition has profound implications for toxicological concerns. As studies with engineered nanoparticles have suggested and investigations of plastic particles are starting to indicate, toxicity induced by micro-nano particles is not only dose-dependent but also related to particle physicochemical characteristics and their effect on cellular interactions and uptake (81, 82). In the case of bottled water from brand C, the cytotoxicity induced by PS nanoplastics plus a small number of PET microplastics would be presumably different from the effect assumed from PET nanoparticles. True comprehensive toxicity evaluation for micro-nano plastics would require multidimensional characterization of plastic particles and the integration of each individual plastic particle regarding their divergent properties on chemical composition and particle morphologies. Single-particle imaging with nanoparticle sensitivity and plastic specificity provides indispensable information to address the rising toxicity concern. Not only it enables plastic particle profiling with accurate exposure quantification, but also it has a unique potential to directly visualize the particle-biology interactions. Therefore, we envision that the data-driven hyperspectral SRS imaging platform will continue bridging the gap of knowledge on plastic pollution at the nano level with an expanded spectral library to study more complicated biological and environmental samples.

## 7. Materials and Methods

### 7.1. Hyperspectral SRS Microscopy.

Hyperspectral SRS imaging is performed under a commercial system constructed by sending a dual-output femtosecond laser system (InSight X3, Spectra-Physics) through an integrated Spectral Focusing Timing and Recombination Unit (SF-TRU, Newport Corporation) (38) and coupled into a multiphoton laser scanning microscope (FVMPE-RS, Olympus). The instrumentation and imaging condition are described in detail in *SI Appendix*.

### 7.2. Sample Preparation.

PS standards of micro-nanospheres in different sizes were bought from Thermo Fisher Invitrogen. Microplastic standards of PET, PP, PE, PVC, and PA were obtained by crushing sub-cm-sized plastic pallets into powders through a freeze mill. Particles suspended in RO water are spread and dried on the surface of the coverslip before being embedded with 1% Agarose gel prepared with D_2_O for SRS imaging. Details are described in *SI Appendix*.

Two bottles of water from the same brand are filtrated through the 0.2-µm pore-sized Anodisc membrane with carefully cleaned glass apparatuses following the procedure described in *SI Appendix*. The harvest membrane is sandwiched according to Fig. 3*B* for SRS imaging. The detailed protocol can be found in *SI Appendix*.

### 7.3. Data Analysis.

The methods for SRS-tailored spectral matching algorithms, synthetic data generation, and automated micro-nano plastic detection are described in detail in *SI Appendix*. The corresponding MATLAB codes are available on GitHub through the following link: https://github.com/qnxcarnation/SRS-tailored-Spectral-Matching-algorithm-for-plastic-identification.git.

## Supplementary Material

Appendix 01 (PDF)

## Data Availability

MATLAB code used for simulation, spectral matching, and plastic analysis; raw imaging data have been deposited in GitHub and Figshare (https://github.com/qnxcarnation/SRS-tailored-Spectral-Matching-algorithm-for-plastic-identification.git (83); and https://doi.org/10.6084/m9.figshare.24635793.v2) (84). All other data are included in the manuscript and/or *SI Appendix*.
