# Supplementary material for: Rapid single-particle chemical imaging of nanoplastics by SRS microscopy
Source: Proc Natl Acad Sci U S A. 2024 Jan 8;121(3):e2300582121. doi: 10.1073/pnas.2300582121 (PMC10801917; doi:10.1073/pnas.2300582121)
Supplement: Supplementary file 1 — Appendix 01 (PDF) [file pnas.2300582121.sapp.pdf]

Supplementary information

Rapid single-particle chemical imaging of nanoplastics by SRS microscopy

Naixin Qian[1], Xin Gao[1], Xiaoqi Lang[1], Huiping Deng[2], Teodora Maria Bratu[2], Qixuan Chen[3], Phoebe Stapleton[4], Beizhan Yan[2*], Wei Min[1,5*]

[1] Department of Chemistry, Columbia University, New York, NY 10027

[2] Lamont-Doherty Earth Observatory of Columbia University, Columbia University, Palisades, NY 10964

[3] Department of Biostatistics, Columbia University Mailman School of Public Health, New York, NY, USA 10032

[4] Department of Pharmacology and Toxicology, Ernest Mario School of Pharmacy, Environmental and Occupational Health Sciences Institute, Rutgers University, New Brunswick, NJ 08854

[5] Department of Biomedical Engineering, Columbia University, New York, NY 10027

[*]Corresponding authors: wm2256@columbia.edu (W.M.) or yanbz@ldeo.columbia.edu (B.Y.)

Table of Contents

I. Materials and Methods

Hyperspectral SRS microscopy

Sample preparation of plastic standards for SRS measurement

SRS-tailored spectral matching algorithms and synthetic data generation

Bottled water sample preparation

Automated micro-nano plastic detection from hyperspectral SRS images

Statistical analysis

II. Supplementary Figures

Figure **S1-9**

III. Supplementary Notes

Supplementary Note 1: Estimation of the number of repetitive units of 100 nm plastics nanoparticles (**Tabel S1**)

Supplementary Note 2: Data-driven learning process for polymer identification (**Tabel S2, Figure S10-12**)

Supplementary Note 3: Quantification analysis using SRS intensity (**Table S3, Figure S13-16**)

Supplementary Note 4: Comparison with other analytical techniques for micro-nanoplastics analysis (**Table S4, Figure S17-18**)

Supplementary Note 5: Nanoplastics exposure extrapolation (**Figure S19**)

Supplementary Note 6: Using fluorescent polystyrene (PS) beads as an internal standard for plastics quantification (**Figure S20**)

I. Materials and Methods

Hyperspectral SRS microscopy

Hyperspectral stimulated Raman scattering microscopy is constructed by sending a dual-output femtosecond laser system (InSight X3, Spectra-Physics) through a commercial Spectral Focusing Timing and Recombination Unit (SF-TRU, Newport Corporation) and coupled into a multiphoton laser scanning microscope (FVMPE-RS, Olympus). The pump beam is tunable in the range of 680-1300 nm, while the Stokes beam is fixed at 1045 nm. Inside SF-TRU, the Stokes beam passes through a resonant electro-optic amplitude modulator (EOM), which modulates the Stokes beam at a 10 MHz resonant frequency. A motorized delay stage (DL125, Newport) is inserted on the Stokes path to adjust the temporal overlap between two beams. A separate grating pair is installed in each beam path for spectral focusing. The pulse width for the pump and the Stokes are calculated to be 4 ps and 3.5 ps, respectively. The existence of grating pairs also reduces the pump tuning range to 790-910 nm (corresponding to a Raman shift range of 1300-3100 cm^{-1}). The two synchronized beams are spatiotemporally overlapped and then coupled into the microscope for SRS imaging. A 25x water objective (XLPlan N, N.A. 1.05, MP, Olympus) is used with a high N.A. condenser lens (oil immersion, N.A. 1.4, Olympus) to collect the pump and stokes beams passing through the samples. A large-area (10 mm x 10 mm) Si photodiode (S3590-09, Hamamatsu) is placed after a telescope to detect pump beam intensity loss after filtering out the Stokes beam with two high-optical-density band-pass filters (FESH0950, Thorlabs). A 64 V DC power supply is used to reverse-biased the photodiode, and the output current of the detector is electronically prefiltered by a band-pass filter (Mini-Circuits, 9.5-11.5 MHz, 50 Ω) before being sent to a fast lock-in amplifier (HF2LI, 50 MHz, Zurich Instruments) with 50 Ω termination for signal demodulation. The in-phase X-output of the lock-in amplifier is fed back into the analog interface box of the microscope to form SRS images. The fast acquisition of the SRS spectrum in each window is achieved by simply adjusting the arrival time of the Stokes beam through a motorized delay stage inside SF-TRU. The delay stage position was calibrated to wavenumber by comparing hyperspectral SRS spectra with spontaneous Raman spectra for polymer standards. Proper calibration is important for consistent matching of the spectrum throughout time. The detailed imaging conditions for different experiments are listed below. All the power is determined after the objective. Unless otherwise mentioned, all SRS images of nanoparticles are acquired with a pixel size of 199 nm.

In the measurement of standard polystyrene (PS) to determine the sensitivity, the central wavelength of the pump beam was 793 nm. Pump power of 120 mW and Stokes power of 214 mW were used for nanosphere measurement and the pixel dwell time settings were in a range of 2 µs - 3x8 µs, with a corresponding time constant from lock-in amplifier to be 2 µs - 6 µs. Lower power (P_{pump}= 60 mW, P_{Stokes}= 129 mW) was used for PS microsphere measurement to avoid burning of the particles with a pixel dwell time and time constant of 2 us, and their ΔI/I ratios were converted to the same power condition of the PS nanosphere imaging using the linear relationship of the beam power and SRS signal. The noise threshold was determined under the imaging condition for measuring PS nanosphere of 130 nm and 240 nm (P_{pump}= 120 mW, P_{Stokes}= 214 mW, the pixel dwell time of 8 µs, time constant 6 µs, averaging 3 times). Resolution is measured by imaging 240 nm PS nanosphere under the same condition with a much smaller pixel size of 16 nm.

In the measurement of library spectra, the central wavelengths we used were 793 nm (spectral window: 3000 cm^{-1} – 3110 cm^{-1}), 804 nm (spectral window: 2800 cm^{-1} – 2980 cm^{-1}), 886 nm (spectral window: 1670 cm^{-1} – 1770 cm^{-1}), and 897 nm (spectral window: 1580 cm^{-1} – 1660 cm^{-1}), with the stepsize on the delay scanning of 0.02 mm, which corresponds to ~3 cm^{-1} converting to the unit of wavenumber. The pump power of P_{pump} = 20 mW and Stokes power of P_{Stokes}= 21-63 mW were used to image micron flakes/fibers of all plastic standards (PE, PP, PA66, PVC, PS, PET, and PMMA), and pump power of P_{pump} = 80 mW and Stokes power of P_{Stokes}= 171 mW were used for *E. coli* sample, which represents possible nonplastic

material from biological components. The pixel dwell time for all the measurement of the library spectrum were 4 μs with a time constant from the lock-in amplifier of 3 μs. All the FOVs are averaged for each polymer material to obtain the corresponding standard SRS spectra used in the library. When measuring the testing data set, a pixel size of 2 um is used in microplastic imaging with scaling and pixel dwell time adjusted to mimic the imaging condition for SRS imaging of nanoplastics.

In the measurement of bottled water samples, P_{pump} = 120 mW and P_{Stokes}= 214 mW were used with a pixel dwell time of 8 μs, time constant of 6 μs, and three times averaging. The central wavelength was the same as the standard measurement with a larger step size of the delay scanning (0.1 mm, ~15 cm^{-1}).

Sample preparation of plastic standards for SRS measurement

PS standards of micro-nanospheres in different sizes were bought from Thermo Fisher Invitrogen (0.1 μm: Lot# 2170524, 0.2 μm: Lot# 2299810, 0.3 μm: Lot# 2344121, 0.5 μm: Lot# 2230856, 0.7 μm: Lot# 2320039, 1 μm Aliphatic Amine latex: batch # 2476-HMD-2,1, 3 μm: Lot# 2145532, 10 μm: Lot# 2392901). Standard PS micro-nanospheres were diluted 100-1000 times with RO water (Sigma Aldrich, Milli-Q) before being spread and dried on the surface of the coverslip with an imaging spacer (Sigma Aldrich, GBL654008). 1% Agarose gel (thermos scientific, Lot# 01162528) was prepared with D_2O (Sigma, 151882-100G) at 95 °C. 5-10 μL of prepared agarose gel was added before the sample was sandwiched between the glass slide and coverslip for SRS imaging.

Microplastic standards of PET, PP, PE, PVC, and PA were obtained from Polymer Kit 1.0 (Hawaii Pacific University). These sub-cm sized plastic pallets were crushed by freeze mill (SPEX SamplePrep 6875D Freezer/Mill Dual Chamber Cryogenic Grinder, 2 rounds for each sample of around 10 pallets, 12 cycles per round with 1 min run time and 1 min cool time at rate 15 CPS per cycle) into powders with irregular particles in micron size. The obtained particles are embedded in the agarose gel described above for SRS imaging.

Another PET standard (GoodFellow, LS567754) used in the spectra library measurement was obtained in fibers with a diameter of 14 microns, which are directly embedded for SRS imaging. PMMA (Sigma Aldrich, 910716, 90875, 73371) and another type of PVC (Sigma Aldrich, 346764), obtained with granules in micron size, are directly embedded for SRS imaging as well.

E· coli cells were fixed with 4% PFA and washed three times before being embedded in the gel for SRS imaging as the control standard representing biomass.

SRS-tailored spectral matching algorithms and synthetic data generation

The calculation of the SMC_{SRS} employed the Optimization Toolbox implemented in Matlab. An SRS spectrum $\boldsymbol{x}$ from detected plastic particles could be decomposed according to **eq.1** in **Figure 2**. The basic premise was that the plastic particle spectrum $\boldsymbol{x}$ should have the same spectral shape as the corresponding normalized standard spectrum $\boldsymbol{s}$ but with different SRS intensity α depending on the particle size. The contribution from the background $\beta\mathbf{b}$ should also be considered. The background spectrum $\mathbf{b}$ was obtained from cropping a particle-free area in the same imaging stack. If the particle size was large enough to fill the focal volume, the contribution from the environmental background $\beta = 0$; In most cases, particles underfilled the focal volume; thus, the contribution of the background is $0 \leq \beta < 1$. The constrained optimization was then applied to find the corresponding α and β so that the reconstructed spectrum $(\alpha\mathbf{s} + \beta\mathbf{b})$ matched the original particle spectrum as well as possible. Mathematically, the optimized solution was the minimal spectral distance $\min_{\alpha, 0 \leq \beta < 1} \|\boldsymbol{x} - (\alpha\mathbf{s} + \beta\mathbf{b})\|_2$, denoted as SMC_{SRS}.

In Matlab, the SRS spectra of plastic nanoparticles were synthesized by randomly scaling the normalized standard spectrum **s** within the range of digital SRS images and applying the estimated noise fluctuation based on our understanding of the instrumentation. Algorithms were developed to mimic the two main noise sources in a typical hyperspectral SRS spectrum. The fundamental noise on the SRS intensity as a shot-noise-limited technique was determined from the intensity distribution of a cropped area in particle images where no particles are detected. A normal distribution was generated based on this intensity distribution and is later applied to the scaled standard spectrum. The possible laser wavelength fluctuation was simulated from the fine-scanned standard spectrum (**Figure 2 A,** ~3 cm^{-1}) by resampling the spectrum in a way to include the local fluctuation for our instrumentation (**Figure S 10**).

Bottled water sample preparation

All the filtration experiments were carried out in a clean lab with fume hood. All glass apparatuses were thoroughly rinsed with chromic acid before filtration to oxidize and remove any organic substances that might cause possible contamination. Then the precleaned apparatuses were then carefully rinsed with the same brand of bottled water to wash away the acid residue and prepare the apparatus for immediate filtration. Water samples were filtered through Al_2O_3 membrane filters (Cytiva Whatman, Anodisc 25 mm, 0.2 µm pore-size). A cover made of clean aluminum foil is always applied to the funnel to minimize possible airborne particulate contamination. Only when filling the water analyte, the cover is carefully removed and the funnel is then quickly filled with the water pour directly out of the original water bottle, after the bottled water was thoroughly shaken to resuspend the possible particles inside. For each membrane sample, two entire bottles of commercial purified water (in the same pack acquired from a large retailer) were filtrated to avoid possible errors introduced by non-uniform subsampling of water analyte within one bottle. After the filtration was finished, the filter was carefully placed in a clean glass petri dish and moved to another room, where the filter was quickly mounted to a coverslip with the upper surface sealed by a trimmed imaging spacer and 1% Agarose gel in 90 µL D_2O. The covered sample was then stored at room temperature in a sealed petri dish with enough D_2O to keep the sample humid by soaking the bottom side of the membrane in the D_2O. Before SRS imaging, the covered sample was mounted on a glass slide, on top of which about 80 µL of D_2O is added to fill the gap between the membrane and the glass slide. Then the excess D_2O on the periphery was wiped out before the application of nail polish on all sides of the coverslip to seal the sample and secure it on the glass slide for hyperspectral SRS imaging. The blank samples were prepared by sandwiching the Anodisc filter in the same way as the bottled water samples described above.

Automated micro-nano plastic detection from hyperspectral SRS images

The obtained hyperspectral SRS images were first aligned in the ImageJ (Plugins: align_slice, https://imagejdocu.list.lu/plugin/aligning/align_slice/start) to correct possible pixel drifts across spectral points. The registered stack of SRS images and a selected region with no particles cropped as the background was then imported into Matlab for further automated image processing. For each type of plastic, possible particle candidates were segmented as regions of interest at the on-resonance images based on the corresponding standard SRS spectrum. Each segmented region of interest first went through a crude screening to make sure there were SRS peaks in the target spectral window. For each obtained crude particle candidate, SMC_{SRS} was then calculated using the optimization algorithms from the corresponding standard spectrum before the threshold (**Supplementary Note 2**) was applied to give a final decision for plastic identification. If the particle is identified to be positive for more than one polymer in the library, the particle will be identified as the polymer with the smallest SMC_{SRS} calculation.

Statistical analysis

For each bottled water sample, 5-8 fields of view were imaged for each sample. For blank analysis, four fields of view were imaged for each sample. At each field of view, duplicates of 5 samples were made and

the average of measures was used to produce reliable quantification statistics. The number of micro-nano particles detected was analyzed using the generalized linear mixed model analysis assuming a Poisson distribution or a negative binomial distribution to account for overdispersion in the count data. The generalized linear mixed models also take into account the correlation between multiple fields of view from the same bottled water sample. Bonferroni correction was used to adjust the significance level of each hypothesis test to control the overall probability of the type I error for multiple hypothesis tests in the statistical conclusions. The value of the adjusted p-values is denoted using *$p<0.05$, **$p<0.01$, ***$p<0.001$. The generalized linear mixed model analyses were conducted using SAS version 9.4 (SAS Institute Inc.).

Supplementary Figures

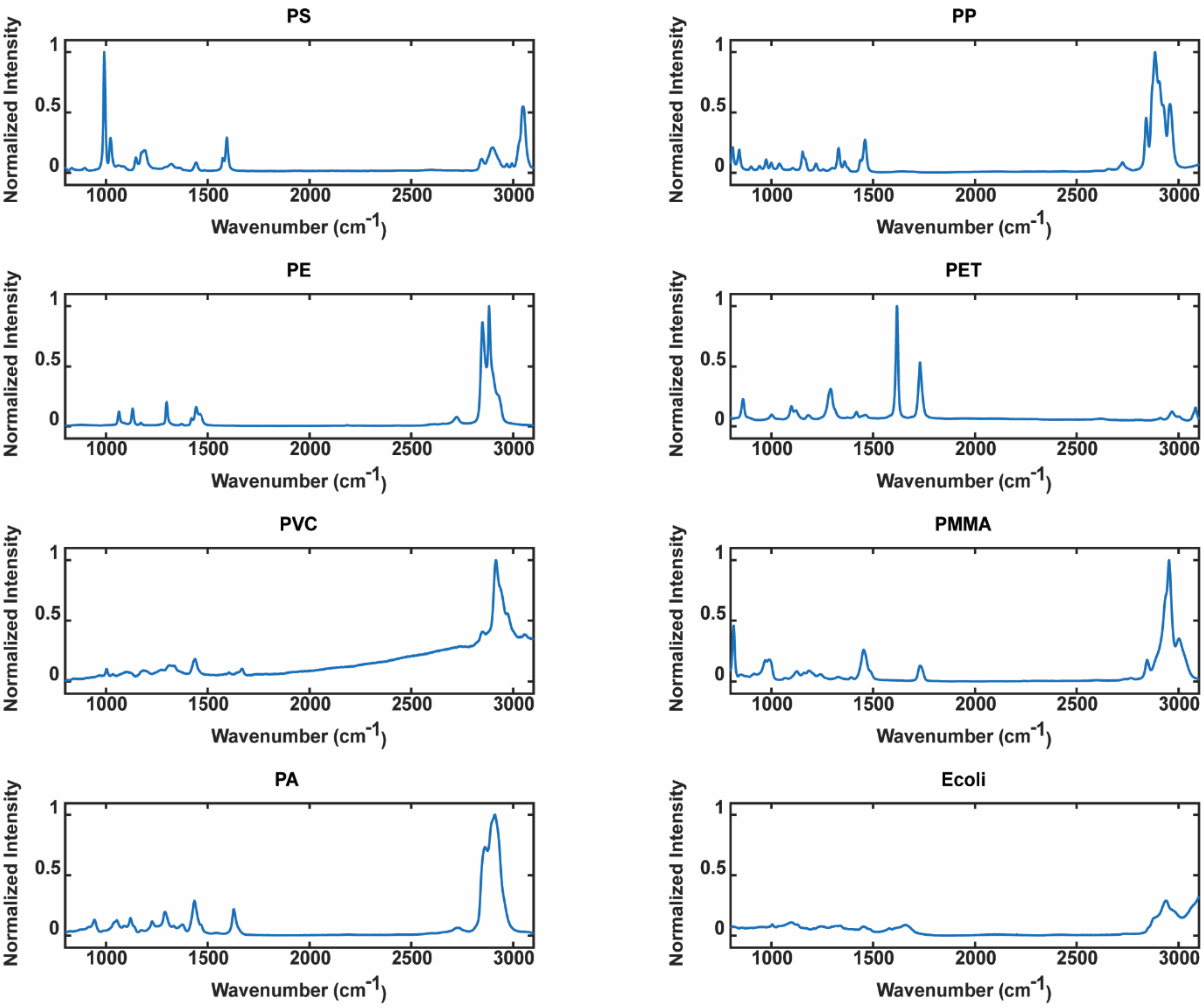

Figure S1. Raman spectra of plastic standards (PS, PP, PE, PET, PVC, PMMA, and PA) and biomass standard (*E. coli*)

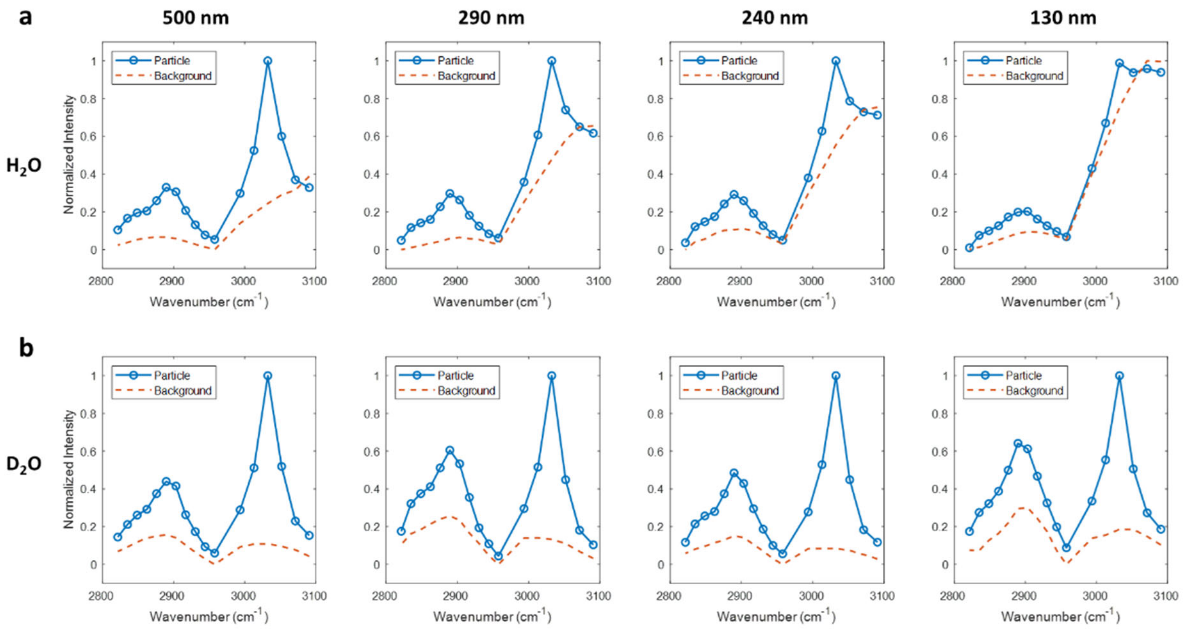

Figure S2. Comparison of SRS spectra of PS nanospheres in agarose gel prepared by a) H_2O or b) D_2O.

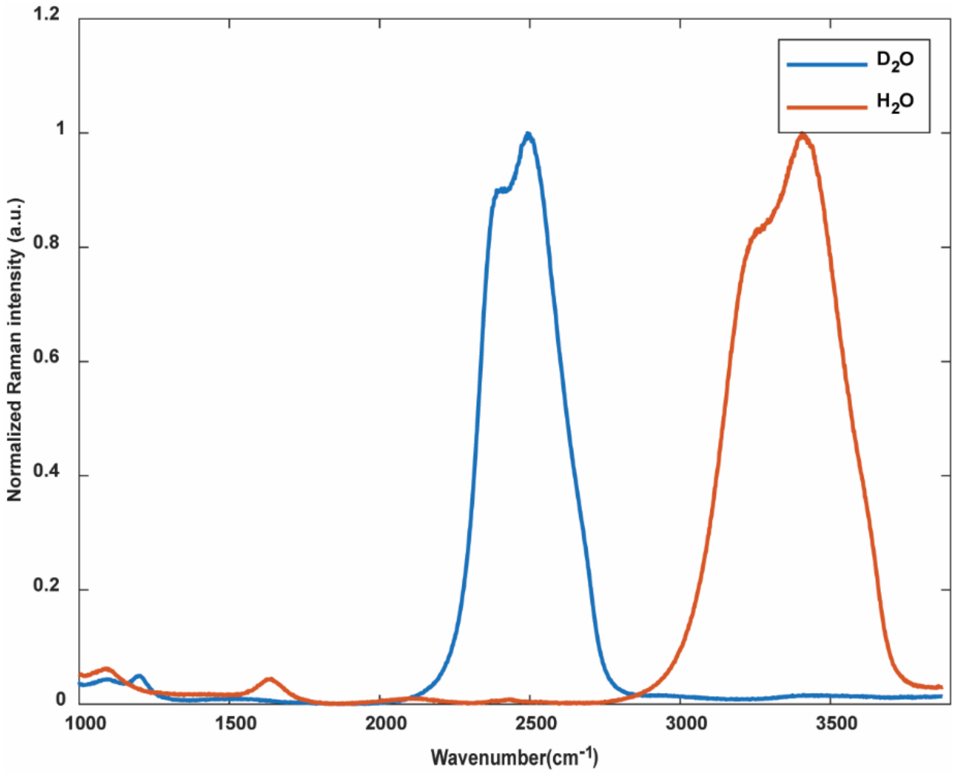

Figure S3. Raman spectra of H_2O and D_2O.

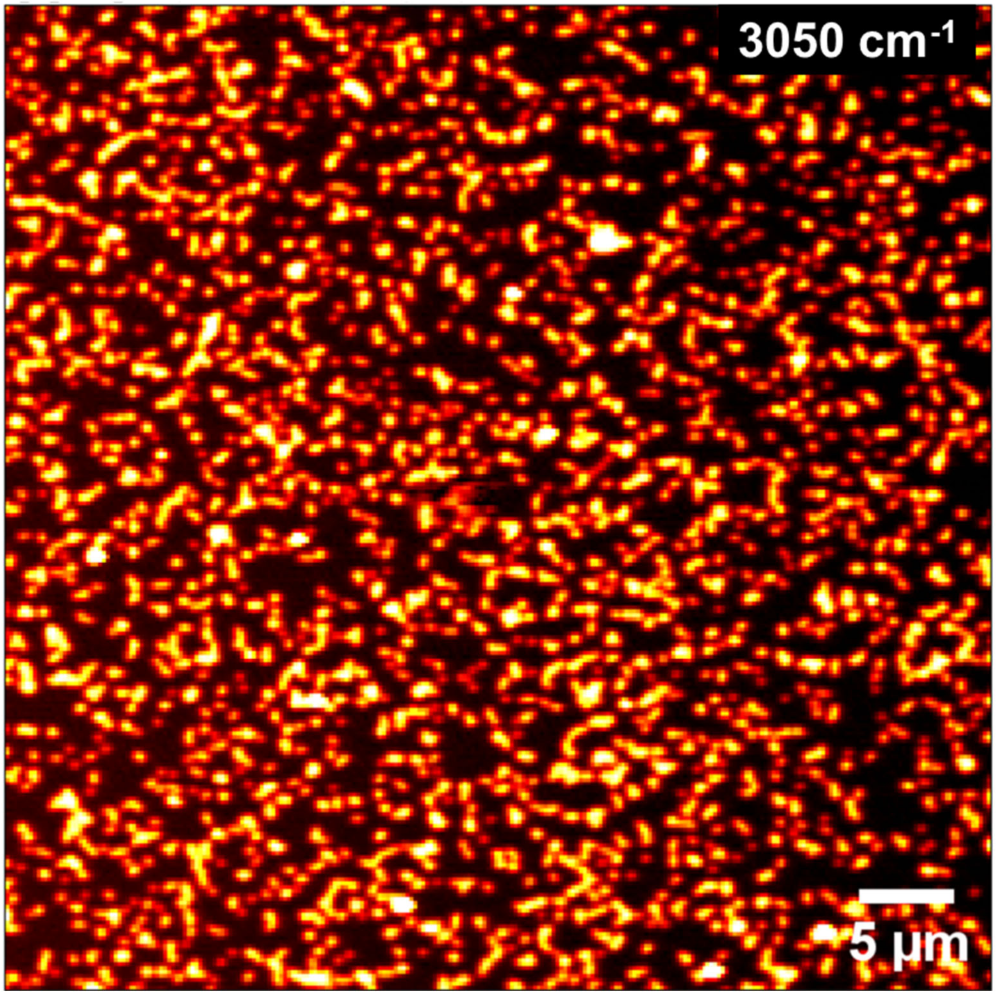

Figure S4. High throughput PS nanosphere measurement enabled by single-channel narrow-band SRS imaging.

Roughly a thousand 240 nm PS nanoparticles in one FOV (51 µm x 51 µm) could be measured under high resolution (pixel size 199 nm) in 1.2 s. Each particle with imaging of a diffraction-limit pattern was analyzed to measure the distribution of SRS intensity for single particles of this specified size. Scale bar: 5 µm.

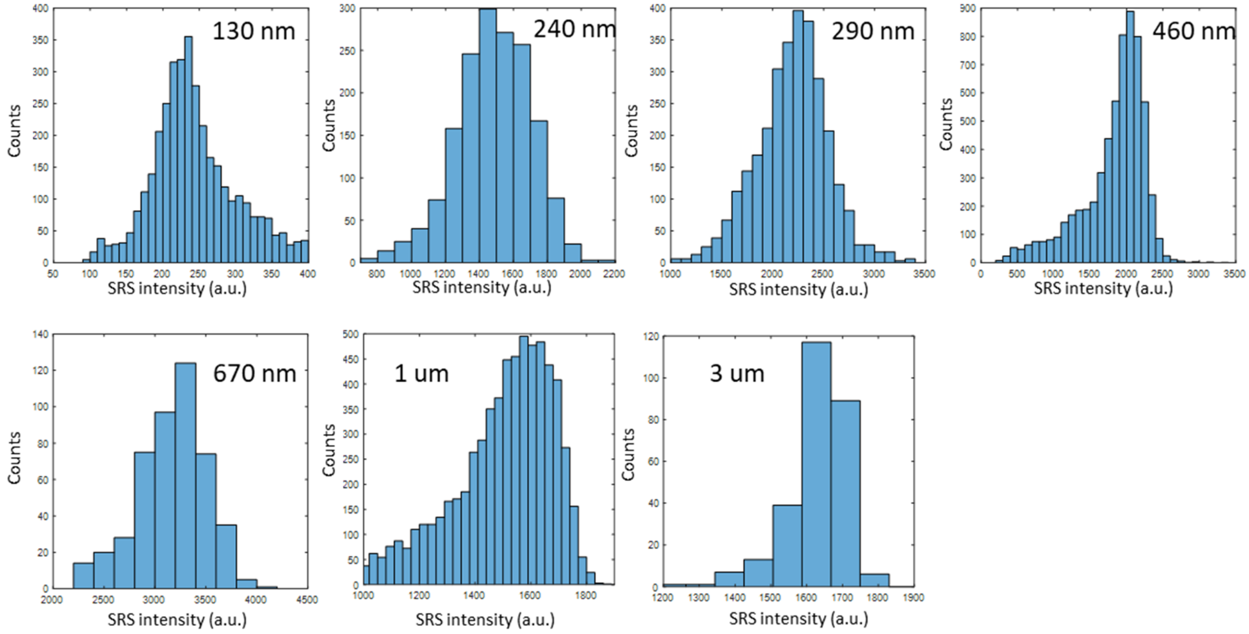

Figure S5. SRS intensity distribution of PS nanospheres with different sizes from single particle imaging.

Figure 1 a-g shows characteristic images for particles with corresponding sizes. For particles below the diffraction limit, only the particles with images of diffraction-limit patterns were counted. The intensity readings for particles of each size were fitted with Gaussian distribution to obtain the mean and standard deviation for analyzing the linear dependence of stimulated Raman loss signals with the particle size in diameter in the log-log scale (**Figure 1 J-K**).

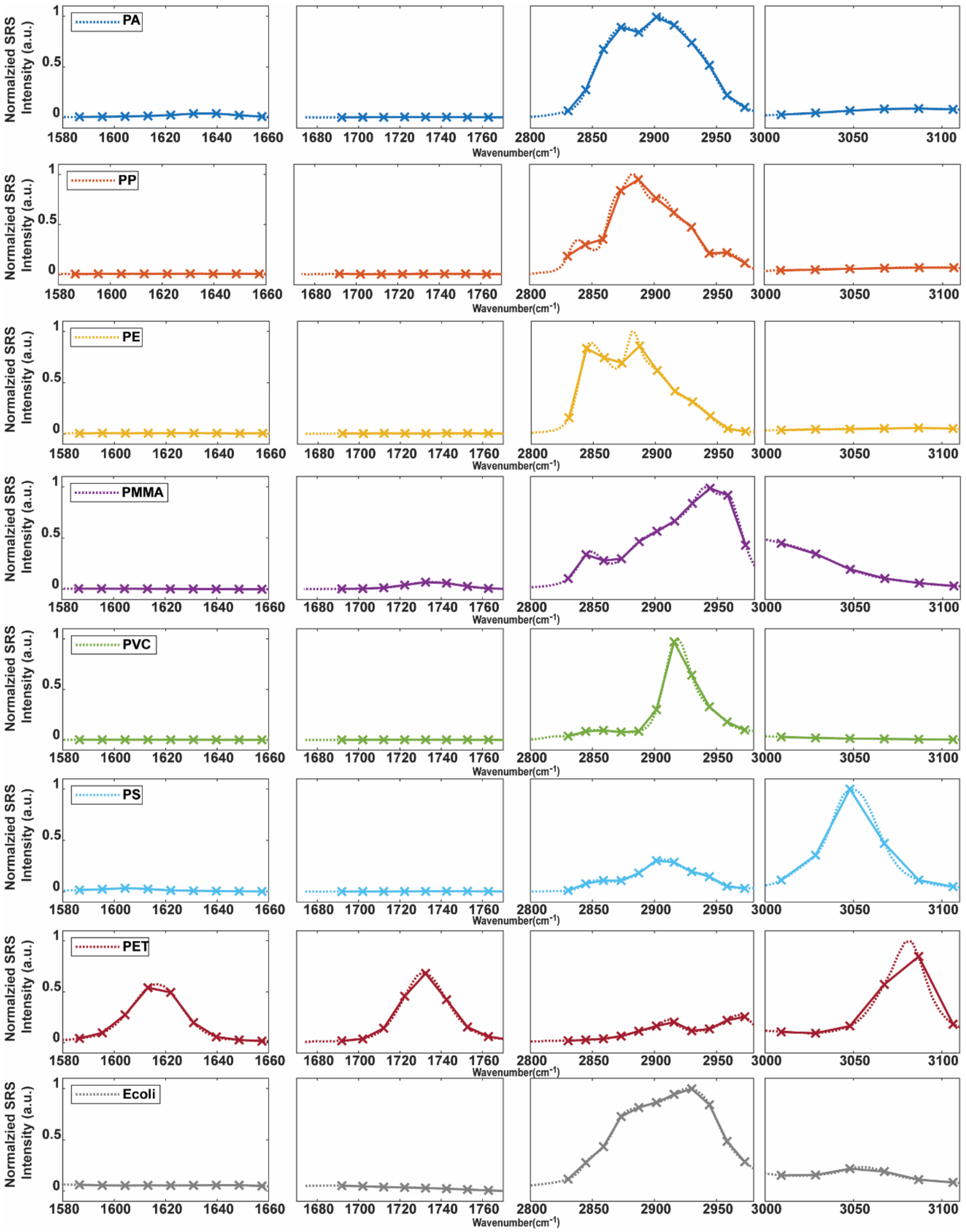

Figure S6. Spectral sampling from the standard library for high-throughput hyperspectral particle imaging with SRS microscopy.

The standard SRS spectra in **Figure 2A** were re-sampled at an interval of 5 spectral points, corresponding to a spectral interval of ~ 15 cm^{-1}. For each spectrum, the corresponding central wavelength for spectral windows from left to right was 897 nm, 886 nm, 804 nm, and 793 nm, respectively. The position was adjusted to best account for the differences in the spectral shape between the spectral standards.

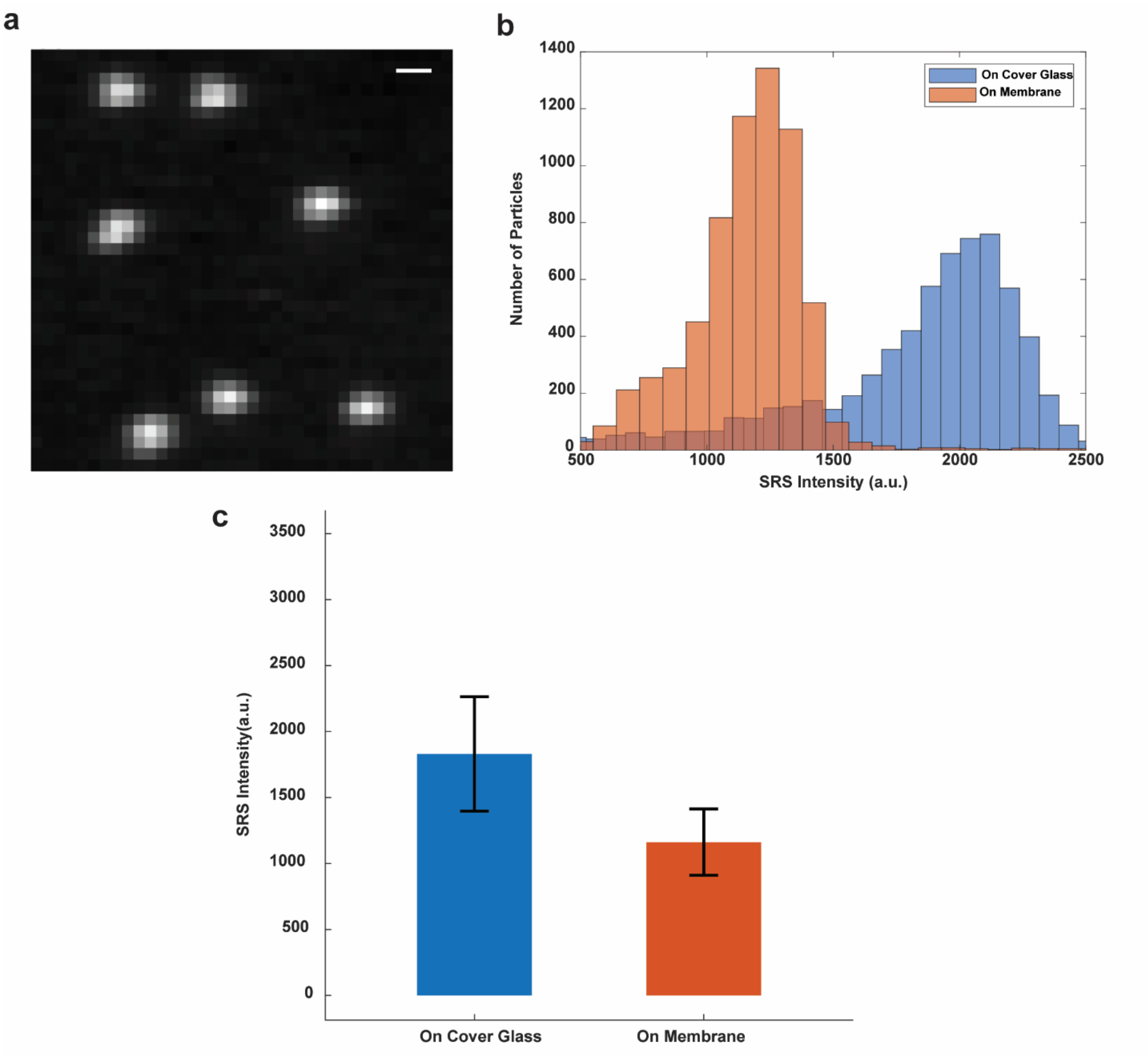

Figure S7. SRS imaging of PS nanospheres on Anodisc aluminum oxide membrane filter

a. Representative SRS images (3050 cm^{-1}) of standard 500 nm PS nanospheres on an Anodisc aluminum oxide membrane filter. The SRS images with and without a filter were identical (**Figure 1 D**), confirming the presence of the filter does not distort the particle images under the transmissive SRS imaging. Scale bar, 0.6 µm; **b.** Distribution of SRS intensity of standard 500 nm PS nanospheres measured in agarose gel on the cover glass and on Anodisc filters, respectively. **c**. The bar chart summarizing the measurement of SRS intensity in **b** indicates ~70% signal retention of the original signal.

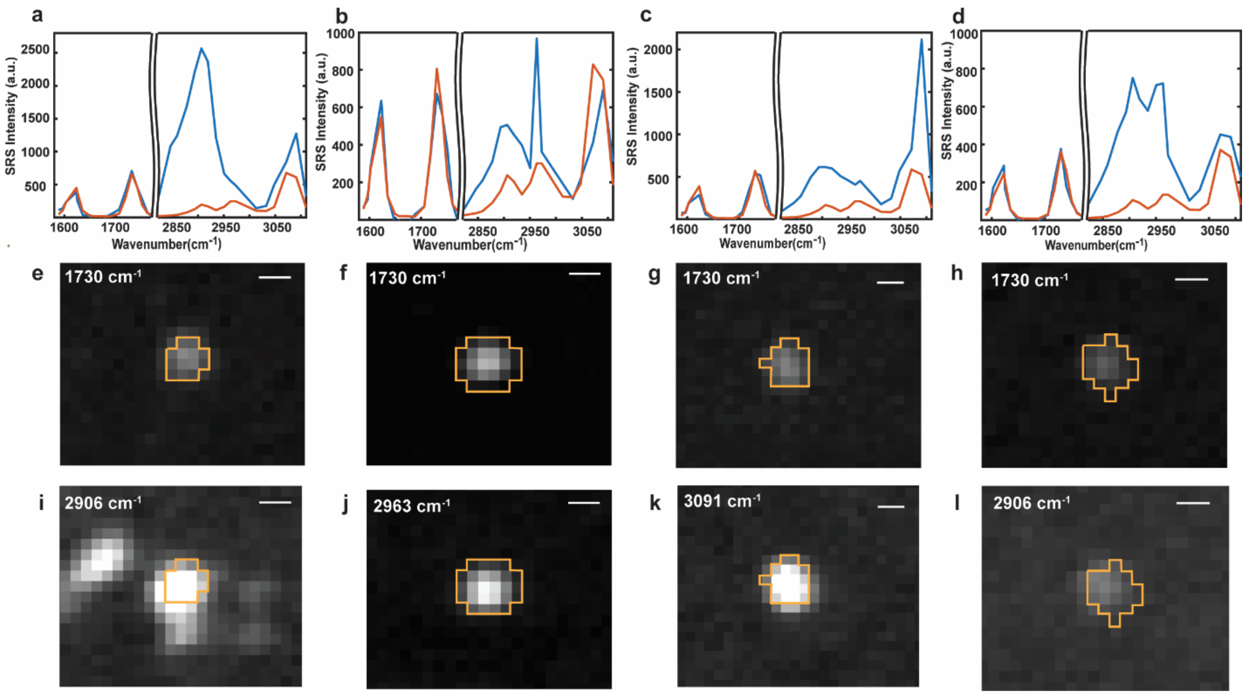

Figure S8. The spectral variation observed for PET particles.

a-d. SRS spectra of the representative PET particles with varied spectral features on the high-frequency C-H region, most likely due to the presence of heteroaggregates. The blue lines are the spectra of detected particles. The orange lines are the correspondingly scaled standard PET spectrum. **e-h.** SRS images of the PET heteroaggregates at C=O vibration (1730 cm^{-1}) corresponding to the spectrum above a-d. **i-l**. SRS images of the PET heteroaggregates at C-H vibration corresponding to the spectrum above a-d. From **Figure S8 a**, **e** , and **i**, spatial chemical heterogeneity *within* the aggregates can be clearly observed as the SRS images at C=O vibration show clear differences from SRS images at C-H vibration. Scale bar, 0.6 µm.

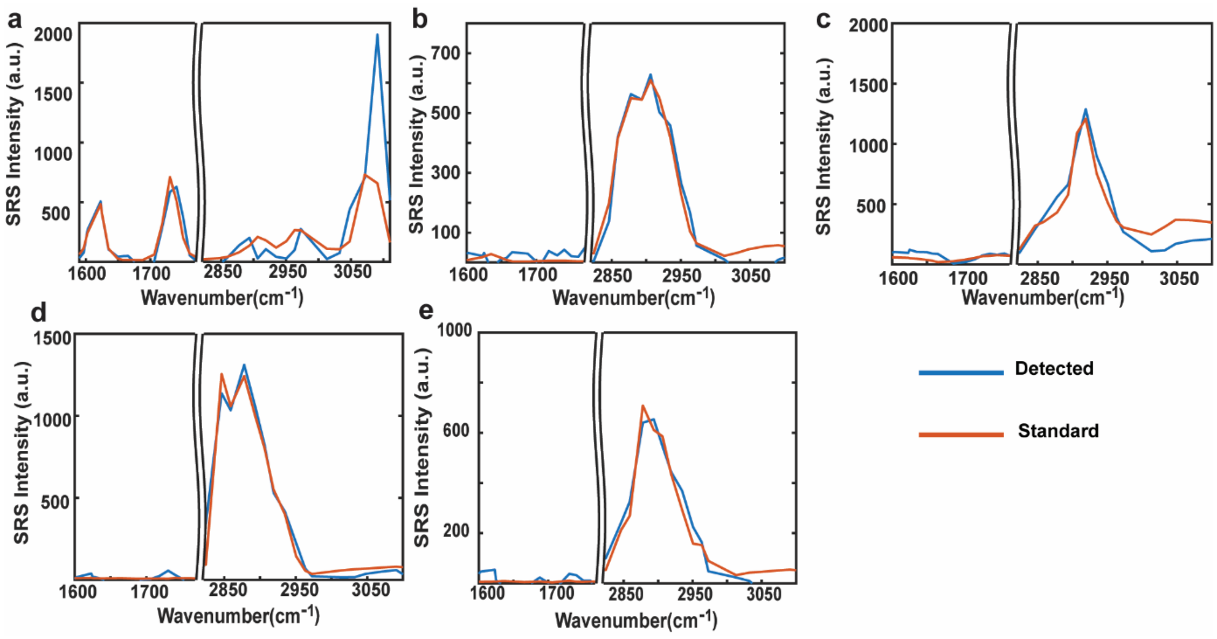

Figure S9. Corresponding SRS spectrum of representative plastic particles with various shapes indicated by different aspect ratios in Figure 6.

a. Corresponding SRS spectrum of the particle in **Figure 6 I**, which is identified as PET**; b.** Corresponding SRS spectrum of the particle in **Figure 6 J**, identified as PA**; c.** Corresponding SRS spectrum of the particle in **Figure 6 K**, identified as PVC**. d.** Corresponding SRS spectrum of the particle in **Figure 6 L** identified as PE**. e.** Corresponding SRS spectrum of the particle in **Figure 6 M**, identified as PP. The blue lines are the spectrum of detected particles. The orange lines are the matched standard spectrum.

Supplementary Notes:

Supplementary Note 1: Estimation of the number of repetitive units of 100 nm plastic nanoparticles

Table S1. Number of Repeating units in 100 nm particles from common plastic types

Plastics polymer	Chemical structure	Unit M.W.	Density (g/cm^3)	100 nm nanoplastics (assuming solid sphere)	
				Mass ($x10^{-16}$ g)	Number of repeating units ($x10^6$)
Polyethylene (PE)	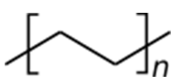	28.05	0.87-0.96<br>0.95*	4.6-5.0	9.8-10.8
Polypropylene (PP)	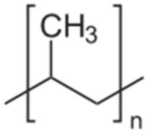	42.08	0.84-0.91<br>0.91*	4.4-4.8	6.3-6.8
Polyamide 66 (PA)	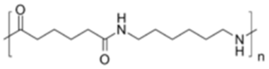	226.32	1.05-1.14<br>1.14*	5.5-6.0	1.5-1.6
Polystyrene (PS)	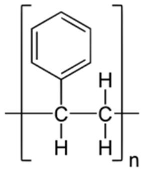	104.15	1.02-1.06<br>1.02*	5.3-5.6	3.1-3.2
Poly(methyl methacrylate) (PMMA)	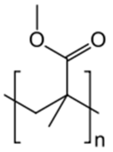	100.12	1.17-1.20<br>1.18*	6.1-6.3	3.7-3.8
Polyvinyl chloride (PVC)	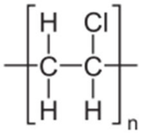	62.50	1.37-1.43<br>1.38*	7.2-7.5	6.9-7.2
Polyethylene terephthalate (PET)	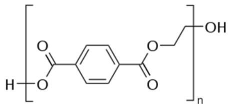	192.17	1.3-1.4<br>1.38*	6.8-7.3	2.1-2.3

All the density information is obtained from ref [1]. For polymers that have a range of density depending on the different forms of existence, * is utilized for the following mass calibration

Supplementary Note 2: Data-driven learning process for polymer identification

Making a nonarbitrary binary judgment for polymer identification requires a learning process based on a large enough dataset. With only PS nanoparticles available as the model, we first explored the simulation condition with the PS nanoparticles. The noise on SRS intensity and the frequency uncertainty imposed by SRS instrument is proposed to be the two key parameters in our model to simulate possible SRS spectra of plastic nanoparticles from the corresponding polymer standards. The intensity fluctuation can be directly measured from the noise in the acquired SRS images. For each FOV measurement, there might be slight changes in the laser profile and the delay stage position, which can result in fluctuation of the actual frequency excited in each measurement around the preset spectral points. Assuming the exact vibrational energy excited at the moment for each measurement follows a Gaussian distribution, the range of uncertainty is modeled by checking the consistency in SMC_{SRS} calculation from the synthetic spectra and measured spectra of PS nanoparticles. A Gaussian profile with FWHM of 10 cm^{-1} is utilized to account for the frequency fluctuation in particle measurements according to the best overlaps between the simulated data and the experimental measurement from the PS nanoparticles (**Figure S10**).

Then the same parameter is used to generate thousands of datapoints for the learning process to find the thresholds for unambiguous identification of all plastic polymers in the library (Described in the methods and pictured in **Figure 2 F**, and **Figure S11**). The threshold condition is determined by fitting a logarithmic function (**a*log(x-c)+b**) that enables the best separation between the target polymer and another polymer in the library that has the closest distribution on the scatter plot.

With the threshold determined for identifying polymer type (**Table S2**), a new set of data with a thousand synthetic data points for each chemical component in the library was generated to evaluate the accuracy of the boundary condition acquired from the learning process. The results after evaluation were summarized in the confusion matrix (**Figure S12**). The performance using SRS-tailored Spectral Matching algorithms with the learning-assisted threshold determination is compared with the performance from conventional spectral matching algorithms based on the simulated data set. For conventional spectral matching algorithms commonly used in the library searching with FTIR and spontaneous Raman, Pearson's Correlation (PC) measurement and Squared Euclidean Cosine (SEC) measurement are presented as the typical examples to make the point. With the measured similarity of 1 to indicate perfect matching, a threshold of 0.7 is most widely used in practice to make a call. Here, with a narrower spectral window and fewer vibrational signatures, the similarity measurement from SRS spectra in the library is generally high (**Figure 2 D,E**). A threshold of 0.7 would create unnecessarily more false positives. We, therefore, optimize the numeric threshold of similarity for SRS spectra matching based on PS nanoparticles, which is the only particle model of nanoplastics available. The threshold is determined to the quartile of PS similarity measurement that ensured a 95% identification rate for PS.

From the confusion matrix, SRS-tailored Spectral Matching algorithms clearly outperform the conventional way of spectral similarity measurement (**Figure S12**), with a maximum of only around 5% misidentification rate in the most challenging case. To further resolve the 5% chance of a particle simultaneously identified as two polymer kinds in the library, we introduce another step in the data analysis workflow to screen for particles that have more than one positive call and identify them as the polymer that gives the smallest SMC_{SRS} value. The performance of the entire identification workflow gives over 96% identification rate for all plastic types and less than 1% false positive rate validated by both the simulation data set (**Figure S12**) and experimental data set (**Figure S12**).

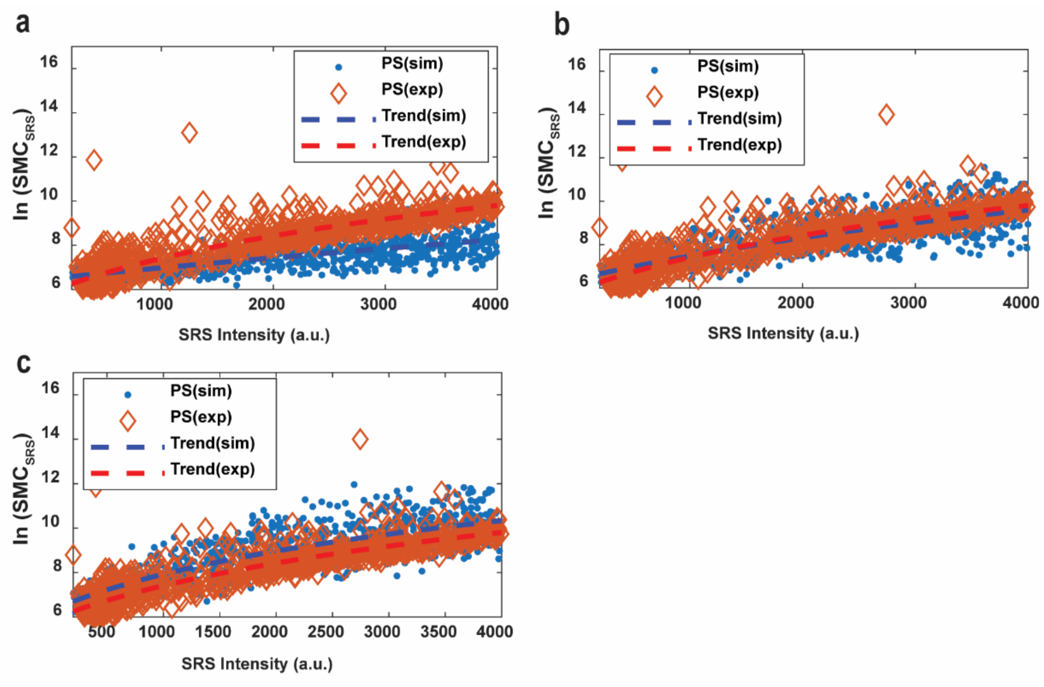

Figure S10. Finding the optimal parameter to simulate frequency uncertainty: A scatter plot of ln(SMC_{SRS}) against SRS intensity α for other plastic standards was generated to compare the experimentally measured PS nanoparticles with simulated PS spectrum on the condition of assuming the instrumental frequency fluctuation is a gaussian distribution with FWHM of **a**) 5 cm^{-1}, **b**) 10 cm^{-1}, **c**) 15 cm^{-1}. Trend lines are fitted with the logarithmic function of a*log(x-c)+b to indicate the trend of the scattered data points.

Tabel S2. Threshold condition determined for polymer identification

Plastics polymer	a	b	c	Minimum Intensity (a.u.)
**PA**	2.39493828359969	-8.84510720757748	-526.399223635400	200
**PP**	2.44961340422964	-9.35922153090780	-544.0442436072430	200
**PE**	2.12960918950470	-5.98617382548675	-260.292871332060	200
**PMMA**	2.15491387069439	-6.04211379746063	-310.263017959368	200
**PVC**	2.29359461274251	-8.20796628579386	-532.177529823619	200
**PS**	2.14297210667609	-6.12510016496094	-343.305676145489	200
**PET**	2.24627056905323	-6.12616605713827	-265.672011592878	200

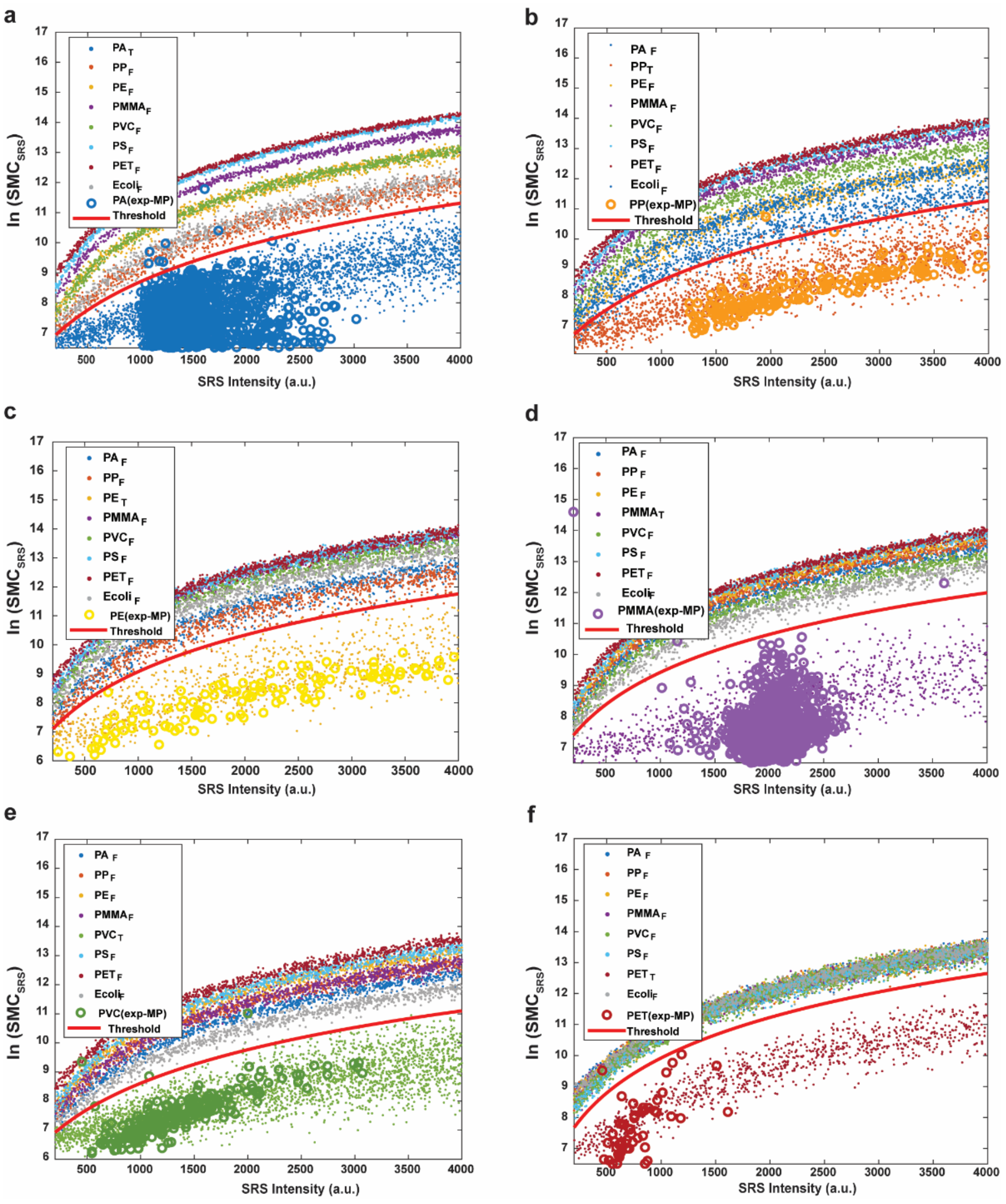

Figure S11. The learning process indicated by scatter plot of ln(SMC$_{SRS}$) against SRS intensity α for other plastic standards: **a**) Polyamide 66 (PA), **b**) Polypropylene (PP), **c**) Polyethylene (PE), **d**) Polymethyl methacrylate (PMMA), **e**) Polyvinyl chloride (PVC), **f**) Polyethylene terephthalate (PET). Data of *E. coli* are also included for comparison. In the legend, the subscript T indicates the synthetic spectral data points of the target polymer in the matching algorithms to evaluate the false negative. The subscript F indicates synthetic spectral data points generated from other polymer types to evaluate the false positive. The red solid lines in each plot are the threshold lines learned from the distribution of labeled synthetic data points. The circular data points are experimental data from hyperspectral SRS imaging of corresponding microplastics.

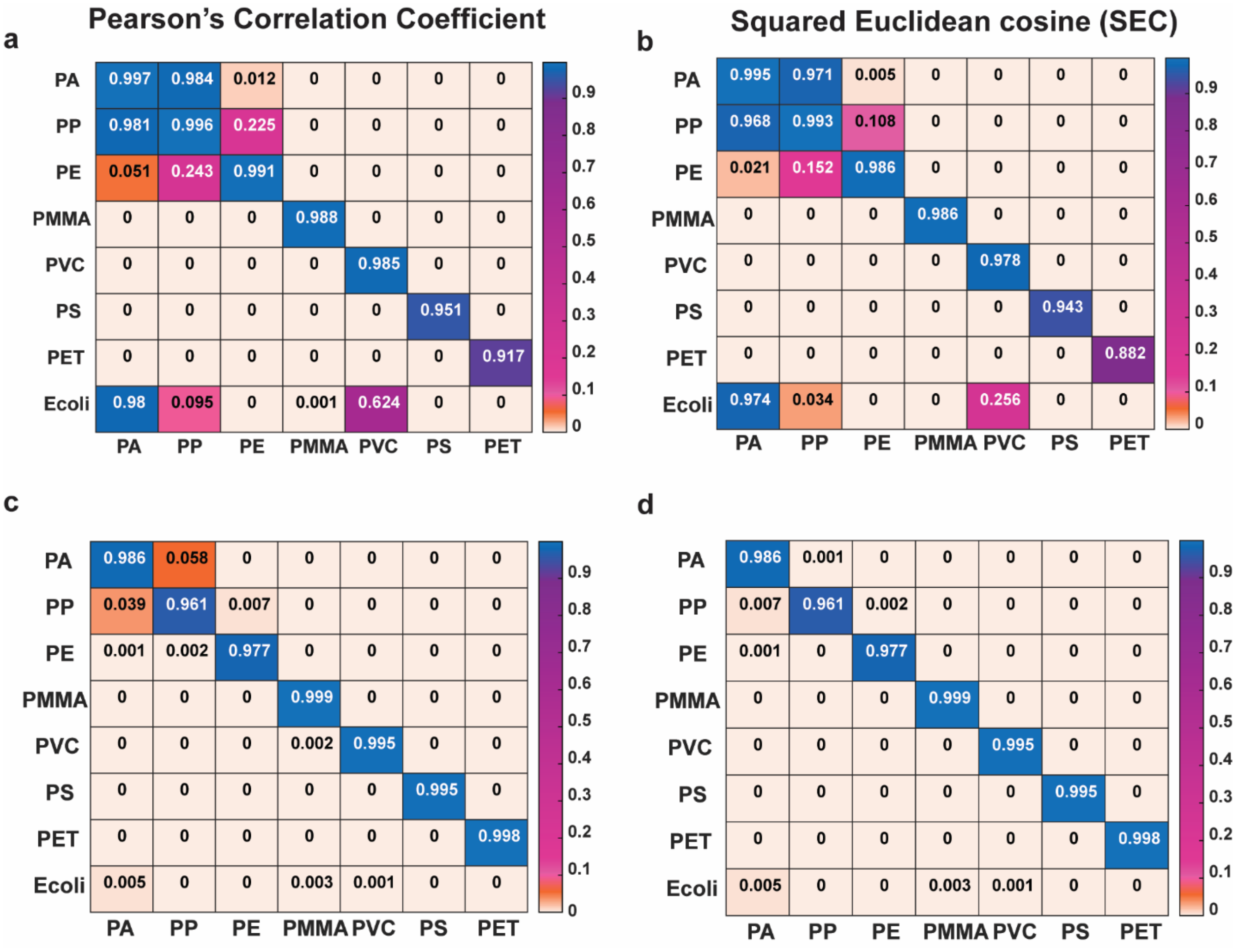

Figure S12. Confusion matrix on plastic identification performance. a) Plastic identification using Pearson's correlation as similarity measurement. **b)** Plastic identification using Squared Euclidean cosine as similarity measurement. **c)** Plastic identification based on SMC_{SRS} measurement with the threshold determined from the data-driven learning process. **d**) Plastic identification based on final data analysis workflow. After applying the determined threshold on SMC_{SRS} measurement for each polymer, particles identified to be with more than one plastic type are re-evaluated to be the polymer that gives the smallest SMC_{SRS} measurement.

Supplementary Note 3: Quantification analysis using SRS intensity

Good linearity between linear stimulated Raman loss signals ($^{\Delta I_p}/_{I_p}$) and the cubic of particle size (slope of 2.97 for the linear trendline fitted under log-log scale) was shown in **Figure 1 J**. To better understand the linear dependence of SRS signal amplitude vs particles' volume for sizes, a simulation is done to further verify the experimental measurement.

Assuming an identical Gaussian profile for both pump and Stokes beams at the focal volume, we first identify the individual beam profile to generate a product of the two beam profiles with FWHM of ~400 nm(**Figure S13 a,b**). The individual beam is found to have an intensity distribution with FWHM of 550 nm(**Figure S13 c,d**).

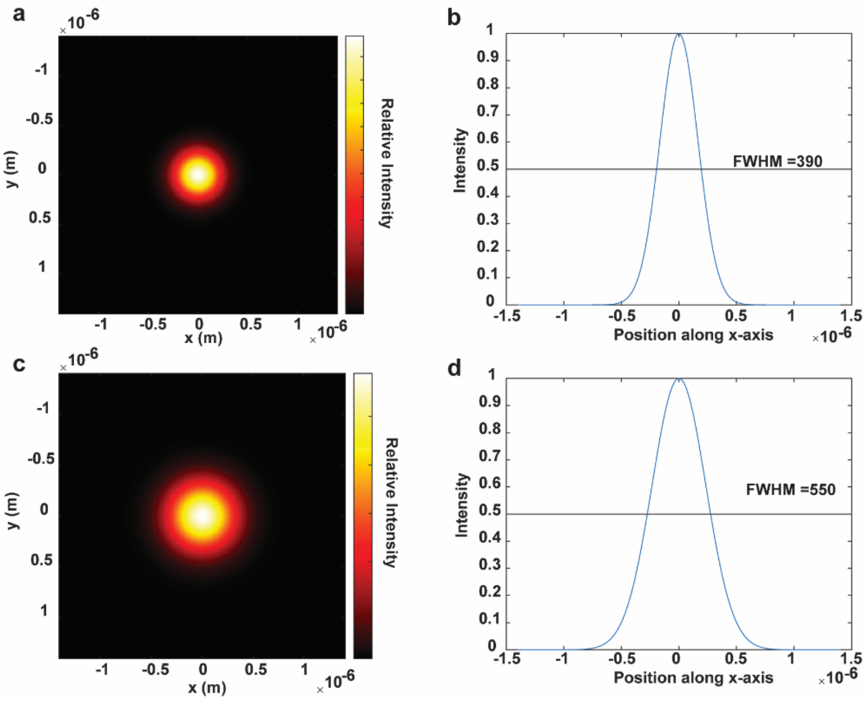

Figure S13. Simulated intensity profile. a) Simulated focal intensity map from the product of the two beams $I_p \cdot I_s$. **b)** The intensity profile suggests a Gaussian distribution with FWHM of ~400 nm, which matches the experimental measurement. **c)** Simulated focal intensity map of individual beam I_p or I_s .**d)** The corresponding intensity profile of individual beam $(I_p\ or\ I_s)$ suggest a Gaussian distribution with the FWHM of 550 nm.

Assuming the PS particles are perfect solid spheres with diameters of the given sizes, we denote the SRS signal of the particle, measured as stimulated Raman loss signals ($^{\Delta I_p}/_{I_p}$), as

$$C \cdot \frac{\int I_p(x,y,z) I_s(x,y,z) S(x,y,z) dV}{\int I_p(x,y,z) dV}$$

$I_p(x,y,z), I_s(x,y,z)$ is the spatial distribution of the pump and Stokes intensity profile in three dimensions (FHWM: 550 nm in x, y dimensions; 1.5 μm in z dimension). $S(x,y,z)$ maps a 3-dimensional solid sphere with a diameter of the given size at the center of the beam. C includes all other constant parameters needed to convert the integration results to the expected ${\Delta I_p}/{I_p}$, which is calculated by dividing the expected SRL for 100 nm PS particles (5×10^{-6}, detailed estimation process in the second section of the main manuscript) by the integral $\frac{\int I_p(x,y,z) I_s(x,y,z) S_{100\ nm}(x,y,z) dV}{\int I_p(x,y,z) dV}$.

Note that in the actual plastic particle imaging experiment, 0.2 μm pixel size is used to enable high-throughput imaging. As most of the PS nanoparticles measured have sizes of a few hundred nanometers, the effect on the signal from continuous galvo scanning during the 200 nm step size should not be ignored. Assuming the galvo continuously scans in the x dimension when measuring particles, the scanning process creates a homogeneous intensity distribution along the x-axis to interact along the particle within this 200 nm sampling step. Taking this into account, we modify the intensity profile (**Figure S14 d**) and simulated the SRS signal(**Figure S14 a-c**). Consistent with our experimental measurement, a good linear dependence of SRS signal amplitude vs particles' volume for sizes up to 0.7 microns is observed. Going above 0.7 μm, SRS signal gradually saturates as the particle fills the focal volume. Full saturation is expected for particles with a size above 1.5 μm, where the particle entirely fills the focal volume in all three dimensions.

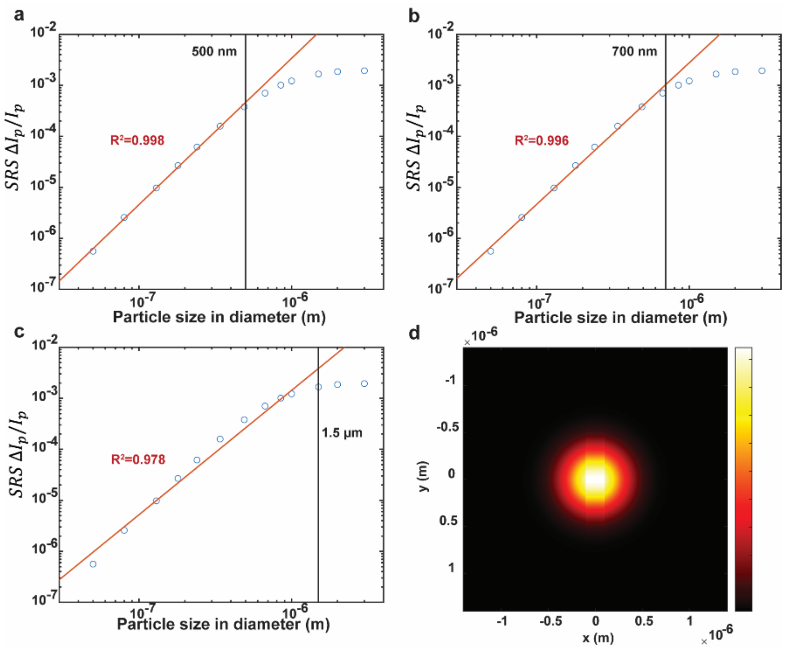

Figure S14. Simulating dependence of SRS signal amplitude vs particles' sizes. a-c) Simulated dependence of SRS signal amplitude vs particles' sizes in diameter plotted in log-log scale. The linear trend line is fitted with data points from particle sizes smaller than **a)** 500 nm, **b)** 700 nm, and **c)** 1.5 μm. A good linear dependence (>0.99) is observed for data points from particle sizes up to 700 nm. **d)** Effected intensity profile of individual beam interacting with particle used in the simulation, considering the continuous galvo scanning behavior across the particle measurement within the 200 nm sampling pixel size.

The validated linear dependence of stimulated Raman loss signals (3050 cm^{-1}) on the cubic of particle size in diameter (μm^3) can be readily used to estimate the size of PS particles below the diffraction limit. Based on the density of PS polymer from **Table S1**, the linear dependence of $\Delta I_p / I_p$ with cubic of size in diameter (μm^3) (**Figure S16 a**) can then be easily converted to the calibration curve for mass estimation of individual particles(**Figure S16 b**):

$$k_{m\_PS} = \frac{k_0}{\frac{\pi}{6} \cdot \rho_{PS}}$$

k_0 – The linear relationship ($y = k_0 x$) of stimulated Raman loss signals with the cubic of particle size in diameter measured from standard PS nanospheres in **Figure S16 a**.

ρ_{PS} – The density of the PS polymer (**Table S1**), which is 1.02 g/cm^3 used in the calculation.

Note that such linearity fundamentally comes from the linear dependency of SRS signal and the concentration of the target analyte, which in principle, can be applied to other plastic polymers. With no standard nanosphere available for direct measurement, we can quantify the relative intensity of other polymers versus PS polymer (RIP). The calibration curve measured from standard PS nanosphere can be transformed easily to characterize both particle size (**Figure S16 a**) and mass (**Figure S16 b**) for other plastic polymers:

$$k_X = k_0 \cdot RIP_X$$

$$k'_{m\_X} = \frac{k_X}{\frac{\pi}{6} \cdot \rho_x}$$

RIP_X - Relative intensity of polymer X vs PS.

ρ_x – The density of the polymer X (**Table S1**).

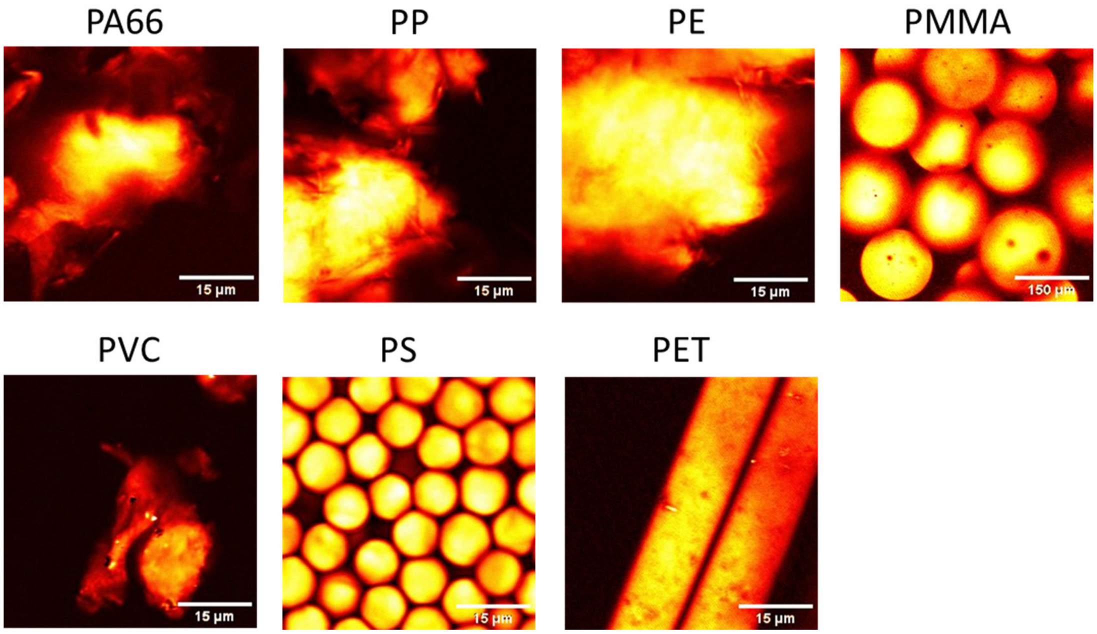

Figure S15. Representative SRS images of plastic standards for each polymer in the library.

The SRS intensity of the plastic standard was measured through imaging at the characteristic peak position of the standard spectrum (**Figure 2 A)**. We notice that for most micron particles obtained from crushing the large pallets by freeze mill, the SRS intensity does not present a uniform distribution within the particle. We reason that the brightest signal comes from the situation where the entire focal volume is filled with the target polymer, while other peripheral regions with dim SRS signals might come from compromised solidity in certain areas of the particles. Therefore, we segmented the brightest areas for each plastic standard to measure the RIP for each polymer (**Table S3**).

Table S3. Relative SRS intensity of plastic polymer standards compared with PS

Plastics polymer	Chemical bonds	Wavenumber	Relative intensity vs PS (RIP)
Polyamide 66 (PA)	sp^3 C-H vibration	2897 cm^{-1}	0.59
Polypropylene (PP)	sp^3 C-H vibration	2881 cm^{-1}	0.94
Polyethylene (PE)	sp^3 C-H vibration	2851 cm^{-1}	0.91
Poly(methyl methacrylate) (PMMA)	sp^3 C-H vibration	2941 cm^{-1}	0.45
Polyvinyl chloride (PVC)	sp^3 C-H vibration	2933 cm^{-1}	0.32
Polystyrene (PS)	sp^2 C-H vibration	3050 cm^{-1}	1
Polyethylene terephthalate (PET)	C=O vibration	1728 cm^{-1}	0.42

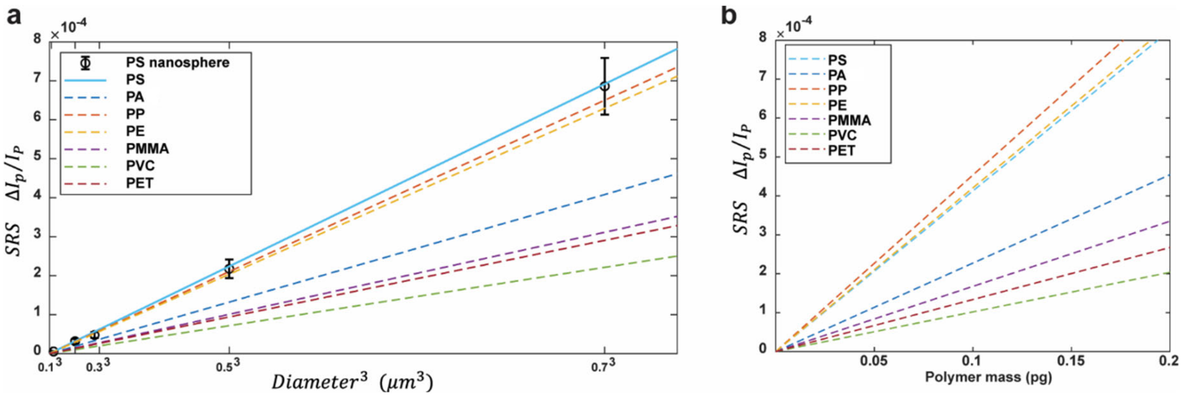

Figure S16. Calibration curves for particle size extrapolation (a) and mass estimation (b) for plastic polymers in the library.

The solid line is converted from the experimental measurement of standard PS nanospheres in **Figure 1J**. The other calibration curves in dashed lines are converted from the calculation described above. Calibration curves in **Figure S16 a** are employed for extrapolation of the particle sizes below the diffraction limits, assuming nanoparticles below the diffraction are a solid nanosphere, whose size is measured in diameter. Calibration curves in **Figure S16 b** are utilized for mass estimation of individual particles, where pixel intensities within the region of interest for each particle are integrated to account for the particles with possible irregular shapes.

Supplementary Note 4: Comparison with other analytical techniques for micro-nanoplastics analysis

Without complicated sample preprocessing, the original composition of the micro-nano plastics in bottled water was preserved to the largest extent. The particle imaging nature of our technique empowers us to analyze plastic particles based on their morphological information. From our result, the analysis of microplastics is readily available with an applied size threshold.

Various techniques have been utilized for micro-nano plastic characterization (**Figure S18**). The capability of detecting plastic particles differs with variations in the specificity, sensitivity, and resolution of applied technologies. Unsurprisingly as a result, the reported levels of microplastics from different analytical methods in bottled water are inconsistent, varying in orders of magnitude (**Table S4**).

To better compare the results with previous studies, we counted the microplastic abundance under different size thresholds (**Figure S17**). In consideration of detection sensitivity and specificity for various techniques utilized in the reported studies, our results on the microplastics abundances were roughly within the same order of magnitude **(Table S4**), confirming the legitimacy of our learning-assisted hyperspectral SRS imaging platform for micro-nano plastics analysis.

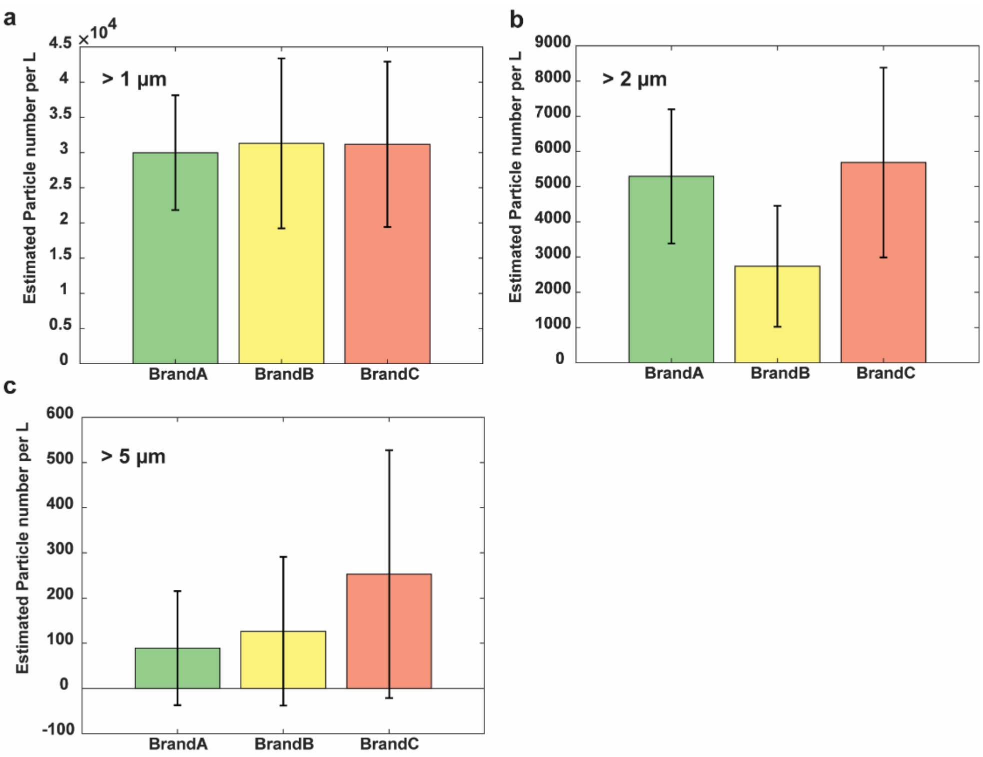

Figure S17. Microplastic abundance in the bottled water.

a-c. The average number of microplastic particles estimated in 1 L of the bottled water. Particles were counted with sizes larger than **a**) 1 µm, **b**) 2 µm, **a**) 5 µm. Error bars, mean ± SEM.

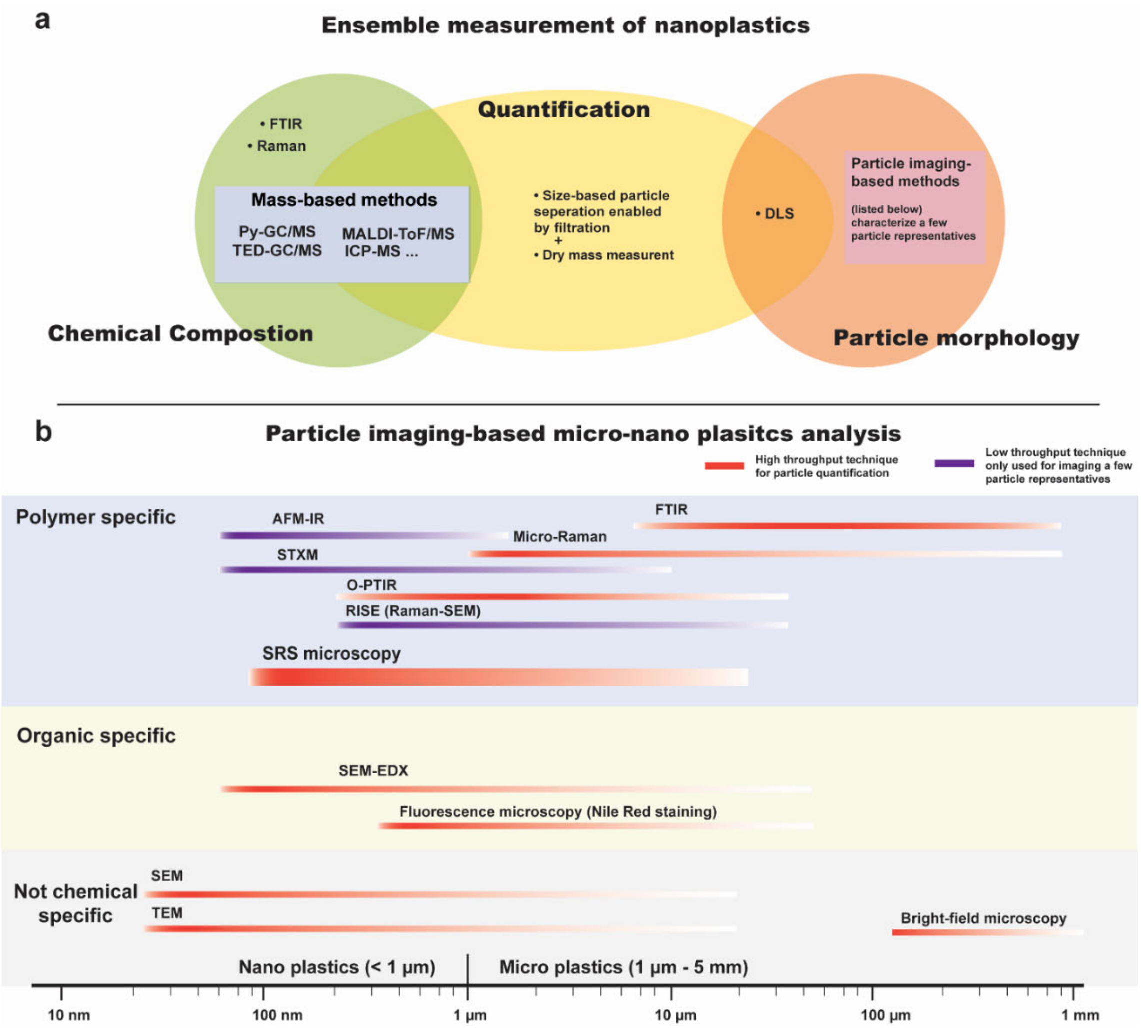

Figure S18. Summary of current techniques on micro-nano plastics analysis

a. Current techniques used in combination to characterize the nanoplastics in an ensembled way. Nanoplastics in the sample are usually first concentrated through filtration with a certain size threshold. Then the quantification is commonly done by measuring the dry mass of the recovered particles before divided into aliquots for different characterization. Chemical composition can be characterized by measuring the FTIR or Raman spectrum of the recovered particle retentates. Mass spectrometry is also commonly used to characterize the chemical composition of the recovered particles and quantification on different polymers can be also obtained with certain optimized methods. For particle morphological characterization, one aliquot is commonly taken for dynamic light scattering (DLS) measurement to map the size distribution of the particles with another aliquot used for particle imaging techniques to show the shape of some representative particles. **b.** Summary of particle imaging-based micro-nano plastic analytical techniques on sensitivity, specificity, and throughput for particle-based quantification. Techniques and representative references are listed here and in **Table S4**: Atomic force microscopy-based infrared spectroscopy (AFM-IR, ref [2]); Scanning transmission X-ray microscopy (STXM, ref [3,4]); Optical-photothermal infrared microspectroscopy (O-PTIR, ref [5]); Correlative Raman Imaging and Scanning Electron Microscopy (RISE, ref [6]); Scanning electron microscopy (SEM, ref [7]); Transmission electron microscopy (TEM, ref [3]).

Year	Technique	Detecting sizes	Abundance (counts/L)	Identification rate	Reference
2018	micro-Raman spectroscopy	$\geq 1\ \mu m$	$(2.6-6.3)\times 10^3$	Not disclosed	8
2018	Nile Red staining + Fourier-transform infrared microscopy (FTIR)	$> 100\ \mu m$	$0-253$	20%	9
	Nile Red staining *	$6.5-100\ \mu m$	$(0-10.3)\times 10^3$	Not apply	
2018	micro-Raman spectroscopy	$\geq 3\ \mu m$	$14-118$	1%	10
2019	Scanning electron microscopy with energy dispersive X-Ray analysis (SEM-EDX)*	$1.28-4.2\ \mu m$	$(3.2-11)\times 10^7$	Not apply	11
2021	FTIR	$\geq 11\ \mu m$	$75-700$	<1%	12
2021	Nile Red staining +FTIR +Raman	$\geq 3\ \mu m$	$52-140$	38%	13

Table S4: Microplastics detection from bottled water in previous studies.

Depending on the techniques employed in different research groups for microplastic analysis, the reported abundances of microplastics in bottled water vary in order of magnitude. As some of the techniques do not contain enough chemical specificity to distinguish between natural organic matter and plastic particles (labeled * in the table above), they tend to report microplastics in orders of magnitude higher than the results from FTIR and micro-Raman. Limited by the resolution, FTIR analysis only includes microplastics that are relatively large in size (> 10 μm) with very low abundance (several hundred counts / L), which is consistent with our results on the large microplastics in **Figure S17 c**. Overall, our results match with the ones reported from micro-Raman analysis in roughly the same order of magnitude (**Figure S17 b**), considering particles of sizes around 1 μm are at the edge of the detection limit for micro-Raman (> 1 μm) and can be easily missed by conventional optical imaging.

Supplementary Note 5: Nanoplastics exposure extrapolation

The number of plastic particles in 1 L of bottled water was estimated from the average number of plastic particles observed in each FOV. The legitimacy of the estimation was based on the following assumptions, which we carefully validated with experiments using standard fluorescent beads.

Assumption 1: Particles uniformly distributed on the membrane surface

Volumetric fluorescence imaging of PS nanospheres indicates a planar particle distribution on the surface of the Anodisc filter membrane (**Figure S19 a**). The particles appear stochastically on the surface plane in a relatively uniform manner across the membrane, as indicated by the distribution of averaged fluorescence intensity of individual field of views on the membrane (**Figure S19 b, c**).

Assumption 2: The actual spreading area of particles is consistent with the circular area of the inner circle of the filtration apparatus (a circular area with a diameter of 13 mm)

The intensity profile of the fluorescence signal across the filter indicates that the circular area where particles concentrate has the diameter of 13 mm (**Figure S19 d**), which is the same diameter as the opening of the glass funnel in the filtration assembly.

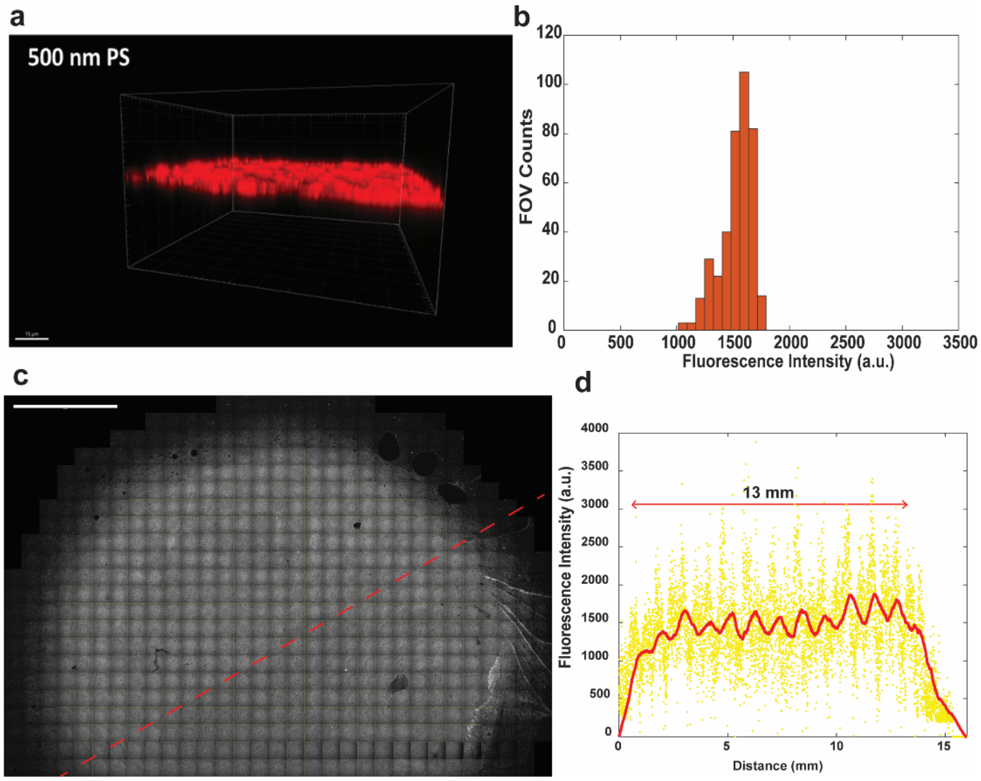

Figure S19. Particle distribution on the Anodisc filter with 0.2 µm pore size indicated by fluorescence PS nanosphere (500 nm).

a. Volumetric fluorescence imaging of PS nanospheres on the membrane. **b**. Histogram plotting the averaged fluorescence intensity measured from individual field of views on the membrane. **c**. Particle distribution of fluorescent PS nanospheres across the entire filter. Scale bar, 3mm. **d**. The intensity profile of the fluorescence signal across the filter. The yellow dots indicate the fluorescence reading from individual pixels across the red dashed line in panel c. The red line indicate the smoothed profile across the membrane.

Supplementary Note 6: Using Fluorescent PS beads as the internal standard for plastics quantification

At the beginning of this work, we attempted to use the fluorescent PS beads as an internal standard for plastic quantification, but the approach was not selected due to discovered contamination issues. Here, we discuss the results and issues when using this internal standard and Milli-Q water, hoping to provide some insights and guidance for other researchers who might want to explore similar things.

Methods

Each bottle/flask of 500 mL water was added with 5 uL 10000x dilution 500 nm yellow-green PS beads (Invitrogen by Thermo Fisher Scientific, Lot# 2295026, ~ 2.9×10^5 particles added per sample). The water was fully mixed and filtrated through the Al_2O_3 membrane filters (Cytiva Whatman, Anodisc 25 mm, 0.2 µm pore-size). After filtering two bottles/flasks, the filter was then embedded the same way in 1% Agarose gel in D_2O for fluorescence and SRS imaging. Fluorescence imaging of the PS internal standard particles was enabled by two-photon fluorescence functionality under the same SRS microscope. The PS particles from bottled water can be distinguished from the PS particles introduced as internal standards through fluorescence imaging. Micro-nano plastics quantifications were extrapolated based on the relative numbers of target plastic particles detected versus the number of internal standard particles detected in the same FOV. For each brand of water, 3 duplicate samples were made (Brand B only 2 samples are imaged), and in each sample, 4 FOVs were sampled for imaging for micro-nano plastic analysis.

Results and Discussion

A. Adding fluorescent particles introduce contamination.

Compared with the data without the addition of internal standard, the average number of particles detected per FOV (**Figure S20 a,** excluding particles as internal standard) was 2-3 times more than the number detected from samples of only bottled water (**Figure 5A**). With exactly the same filtration process and bottled water samples, we reasoned the extra particles come from internal standards added to the bottled water samples. The contamination was so overwhelming that the exposure of micro-nano plastics from bottled water is hard to be analyzed by comparing the blank samples of RO water from the Milli-Q system (CDUFBI001). Eventually, we discarded the idea of adding any internal standard to prevent introducing contamination and extrapolated the plastic particle exposure based on the area under the assumption described in **Supplementary Note 5**. This failed experiment also teach us a lesson on the very liability to introduce contamination upon adding any external reagents to the samples for nanoplastics analysis.

B. RO water from the Milli-Q system is not a perfect blank for nanoplastics analysis.

MilliQ water seems to have the same level of plastic contamination compared with bottled water measured (**Figure S20 a**). Particularly, there were actually more polyamide plastic particles detected from the Milli-Q water than one of the brands of bottled water (**Figure S20 d**). Despite the contamination issue, the qualitative comparison gives a consistent result to the one without internal standard (**Figure 5 B**): Brand A is the brand with the most PA particles (**Figure S20 d**), Brand B is the brand with the most PVC particles (**Figure S20 b**), and Brand C is the brand with most PET particles (**Figure S20 c**) among the analysis comparing three different brands of bottled water. Therefore, we started to question whether Milli-Q water, the golden blank for microplastic analysis, is still the perfect blank when it comes to nanoplastic analysis. Plastics are major components in many parts of the entire water purification system. Especially, polyamides are the most common material used as the RO membranes widely adopted in water treatment. Since the system is not specially designed for nanoplastic analysis, our results suggest that it is very likely Milli-Q

water contains nanoplastics and can no longer be used as the lab blank for nanoplastic studies. To make sure the blank samples taken as reference are with minimize contamination, we eventually used Anodisc Al_2O_3 membrane filters as the blank for bottled water analysis this time. We believe more efforts are needed to establish a contamination-free water sample in the future as a perfect blank sample for procedure control.

C. Estimation from internal standards supported the legitimacy of direct particle extrapolation based on area.

No PET particles were found in the Milli-Q water sample with the internal standard, making PET analysis in the bottled water possible with no concern about contamination from the internal standard, Milli-Q water, or airborne plastic particles. The estimated number of PET particles in 1 L bottled water was on the order of 10^4-10^5, which is consistent with the results estimated from extrapolation based on the area (**Figure 5 C, D**). Such consistency from another angle supported the legitimacy of direct particle extrapolation methods for micro-nano plastics quantification.

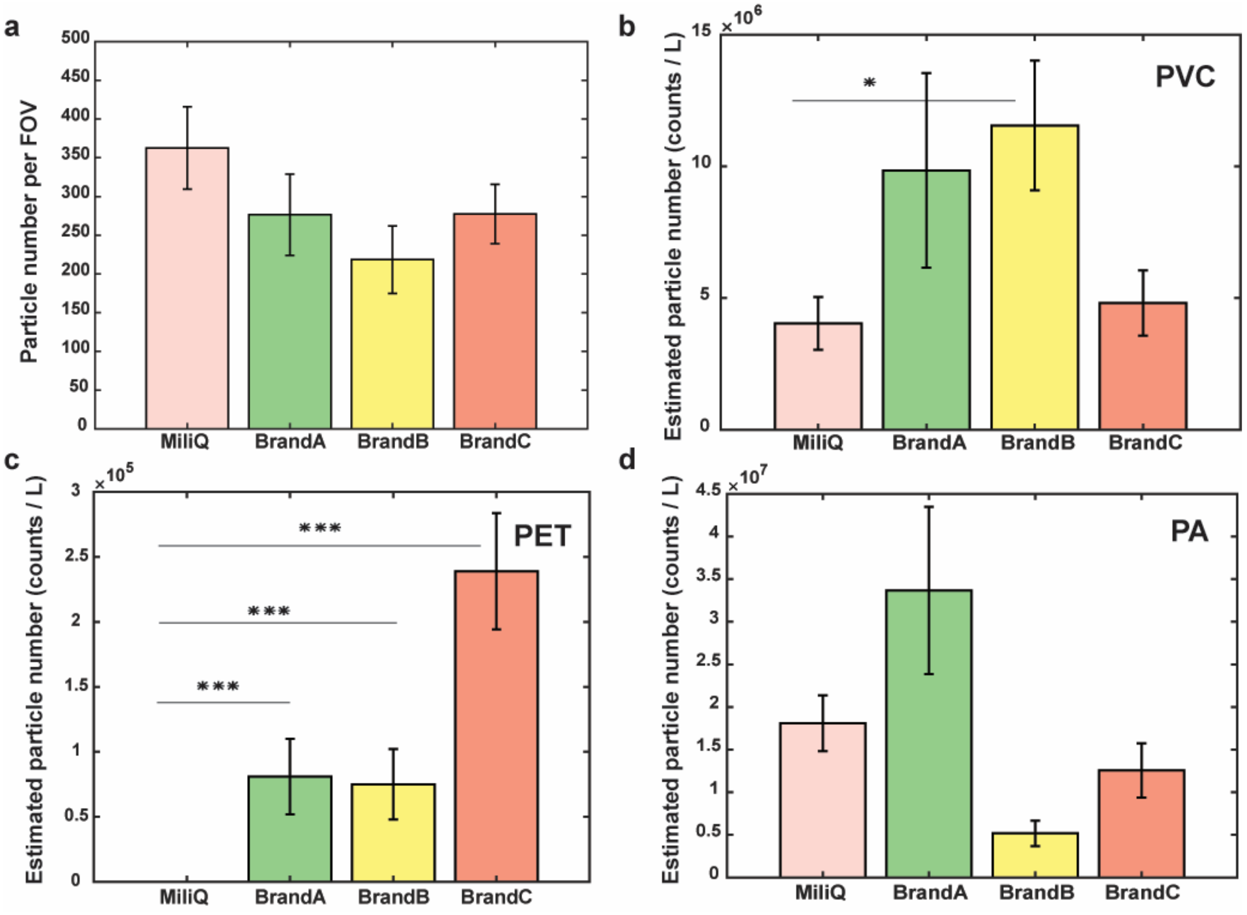

Figure S20. Micro-nano plastics quantification from analysis using fluorescent PS beads as the internal standard. a. The average number of plastic particles detected in one field of view. Error bars, mean ± SEM. **b.** The number of PVC plastic particles estimated in 1 L of bottled water. Error bars, mean ± SEM. **c**. The number of PET plastic particles estimated in 1 L of bottled water. Error bars, mean ± SEM. **d**. The number of polyamide plastic particles estimated in 1 L of bottled water. Error bars, mean ± SEM. Statistically significant differences were determined using generalized linear mixed model analysis with Bonferroni correction. *$p<0.05$, **$p<0.01$, ***$p<0.001$.

Supplementary References

1. Wypych, G., *Handbook of polymers*. Elsevier: 2022.
2. Dazzi, A.; Prater, C. B., AFM-IR: Technology and applications in nanoscale infrared spectroscopy and chemical imaging. *Chemical reviews* **2017,** *117* (7), 5146-5173.
3. Yang, T.; Luo, J.; Nowack, B., Characterization of nanoplastics, fibrils, and microplastics released during washing and abrasion of polyester textiles. *Environmental Science & Technology* **2021,** *55* (23), 15873-15881.
4. Foetisch, A.; Filella, M.; Watts, B.; Vinot, L. H.; Bigalke, M., Identification and characterisation of individual nanoplastics by scanning transmission X-ray microscopy (STXM). *J Hazard Mater* **2022,** *426*, 127804.
5. Su, Y.; Hu, X.; Tang, H.; Lu, K.; Li, H.; Liu, S.; Xing, B.; Ji, R., Steam disinfection releases micro(nano)plastics from silicone-rubber baby teats as examined by optical photothermal infrared microspectroscopy. *Nat Nanotechnol* **2022,** *17* (1), 76-85.
6. Zhang, W.; Dong, Z.; Zhu, L.; Hou, Y.; Qiu, Y., Direct observation of the release of nanoplastics from commercially recycled plastics with correlative Raman imaging and scanning electron microscopy. *Acs Nano* **2020,** *14* (7), 7920-7926.
7. Hernandez, L. M.; Xu, E. G.; Larsson, H. C.; Tahara, R.; Maisuria, V. B.; Tufenkji, N., Plastic teabags release billions of microparticles and nanoparticles into tea. *Environmental science & technology* **2019,** *53* (21), 12300-12310.
8. Ossmann, B. E.; Sarau, G.; Holtmannspotter, H.; Pischetsrieder, M.; Christiansen, S. H.; Dicke, W., Small-sized microplastics and pigmented particles in bottled mineral water. *Water Res* **2018,** *141*, 307-316.
9. Mason, S. A.; Welch, V. G.; Neratko, J., Synthetic polymer contamination in bottled water. *Frontiers in chemistry* **2018**, 407.
10. Schymanski, D.; Goldbeck, C.; Humpf, H.-U.; Fürst, P., Analysis of microplastics in water by micro-Raman spectroscopy: release of plastic particles from different packaging into mineral water. *Water research* **2018,** *129*, 154-162.
11. Zuccarello, P.; Ferrante, M.; Cristaldi, A.; Copat, C.; Grasso, A.; Sangregorio, D.; Fiore, M.; Oliveri Conti, G., Exposure to microplastics (<10mum) associated to plastic bottles mineral water consumption: The first quantitative study. *Water Res* **2019,** *157*, 365-371.
12. Weisser, J.; Beer, I.; Hufnagl, B.; Hofmann, T.; Lohninger, H.; Ivleva, N. P.; Glas, K., From the well to the bottle: identifying sources of microplastics in mineral water. *Water* **2021,** *13* (6), 841.
13. Kankanige, D.; Babel, S., Smaller-sized micro-plastics (MPs) contamination in single-use PET-bottled water in Thailand. *Science of the total environment* **2020,** *717*, 137232.
